# The microbiota maintain homeostasis of liver-resident γδT-17 cells in a lipid antigen/CD1d-dependent manner

**DOI:** 10.1038/ncomms13839

**Published:** 2017-01-09

**Authors:** Fenglei Li, Xiaolei Hao, Yongyan Chen, Li Bai, Xiang Gao, Zhexiong Lian, Haiming Wei, Rui Sun, Zhigang Tian

**Affiliations:** 1 Institute of Immunology and the Key Laboratory of Innate Immunity and Chronic Disease (Chinese Academy of Science), School of Life Science and Medical Center, University of Science and Technology of China, Hefei 230027, China; 2 Hefei National Laboratory for Physical Sciences at Microscale, Hefei, Anhui 230027, China; 3 Model Animal Research Center, Nanjing University, Nanjing, Jiangsu 210061, China; 4 Collaborative Innovation Center for Diagnosis and Treatment of Infectious Diseases, State Key Laboratory for Diagnosis and Treatment of Infectious Diseases, First Affiliated Hospital, College of Medicine, Zhejiang University, Hangzhou, Zhejiang 310003, China

## Abstract

The microbiota control regional immunity using mechanisms such as inducing IL-17A-producing γδ T (γδT-17) cells in various tissues. However, little is known regarding hepatic γδT cells that are constantly stimulated by gut commensal microbes. Here we show hepatic γδT cells are liver-resident cells and predominant producers of IL-17A. The microbiota sustain hepatic γδT-17 cell homeostasis, including activation, survival and proliferation. The global commensal quantity affects the number of liver-resident γδT-17 cells; indeed, *E. coli* alone can generate γδT-17 cells in a dose-dependent manner. Liver-resident γδT-17 cell homeostasis depends on hepatocyte-expressed CD1d, that present lipid antigen, but not Toll-like receptors or IL-1/IL-23 receptor signalling. Supplementing mice *in vivo* or loading hepatocytes *in vitro* with exogenous commensal lipid antigens augments the hepatic γδT-17 cell number. Moreover, the microbiota accelerate nonalcoholic fatty liver disease through hepatic γδT-17 cells. Thus, our work describes a unique liver-resident γδT-17 cell subset maintained by gut commensal microbes through CD1d/lipid antigens.

## Abstract (web summary)

γδ T cells are major producers of IL-17A in response to microbial infection. Here the authors show that a high load of commensal microbes can maintain homeostasis of IL-17A^+^ γδ T cells in the liver via CD1d antigen presentation, with implications for liver diseases.

The liver is situated in a unique systemic circulation system that receives blood from both the hepatic artery and the portal vein, making this organ a prime location for both metabolic and immune function^1^,^2^,^3^. However, the precise mechanism that connects the microbiota and the hepatic immune response is seldom reported. Bacterial translocation and pathogen-associated molecular pattern (PAMP) transport are the two main events that have been observed in the liver–gut axis^4^,^5^. However, the proposed mechanisms will remain elusive until the soluble factors from the microbiota and their cellular targets in liver-gut axis are determined.

The liver is enriched in innate immune cells, including γδT cells at a frequency of 3–5% (5 to 10-fold greater than in other tissues or organs) within total liver lymphocytes^1^. γδT cells function as a bridge between innate and adaptive immunity because they express a rearranged T-cell receptor (TCR) that recognizes certain antigens and can also rapidly secrete pro-inflammatory cytokines including interleukin (IL)-17A upon stimulation^6^. By producing IL-17A to recruit neutrophils and enhance adaptive immunity, IL-17A-producing γδT (γδT-17) cells have an important role in host defence against bacterial, fungal and viral infections, as well as stress, tumour surveillance and autoimmune diseases^7^. However, although hepatic γδT cells are involved in several liver immune diseases^8^, their physiological characteristics, and why the liver contains such high levels of γδT cells, are unknown.

CD1d, a typical lipid presentation molecule for natural killer T (NKT) cells^9^, can also present lipid antigens to the γδTCR and activate γδT cells^10^. A γδT cell subset in human blood can respond to CD1d-presented sulfatide, a lipid antigen present in both hosts and bacteria^11^. Another γδT cell subset in the mouse duodenum can respond to exogenous lipid antigens including phosphatidylcholine, phosphatidylethanolamine (PE) and phosphatidylglycerol (PG) presented by CD1d^12^. The liver constantly encounters microbial lipid components, and crosstalk occurs between CD1d and liver NKT cells^13^,^14^,^15^,^16^; however, little is known regarding the role of γδT cells in this process.

Here we compare γδT cells originating from several organs and identify a liver-resident γδT-cell population that predominantly produces IL-17A. The microbiota maintain hepatic γδT-17 cell homeostasis, the underlying mechanism of which involves microbiota lipid antigens presented by hepatocyte-expressed CD1d, but not PAMPs or cytokine signals. Moreover, liver-resident γδT cells responding to the microbiota contribute to nonalcoholic fatty liver disease (NAFLD).

## Results

### Hepatic γδT cells produce IL-17A

**Figure 1:**
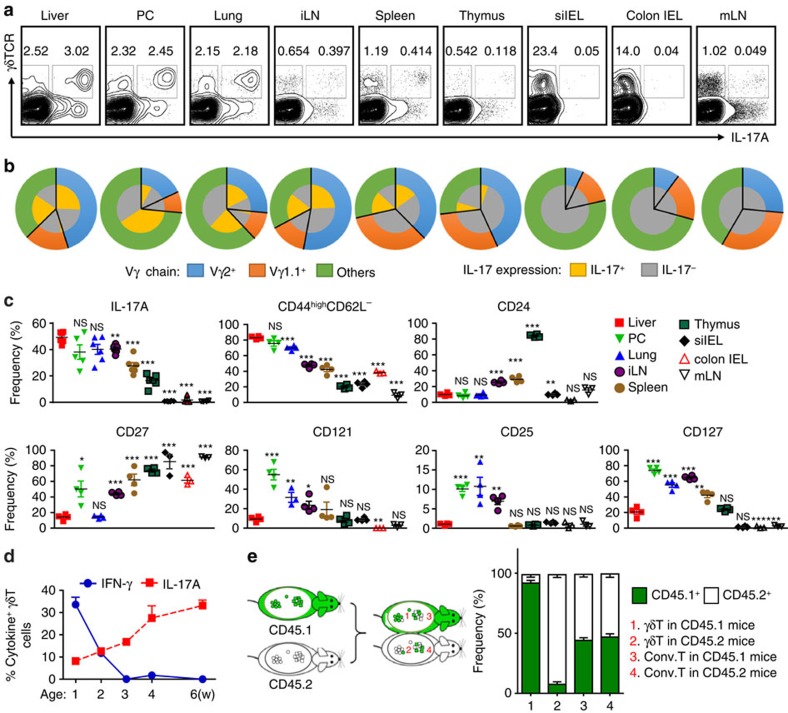
Hepatic γδT-17 cells are major γδT population and liver-resident in adults. (**a**) FACS analysis of IL-17A expression by PMA/ionomycin-stimulated γδT cells from the indicated organs of B6 mice, gated on CD3^+^ T cells. (**b**) FACS analysis of Vγ chain usage and IL-17A expression by each γδT-cell subtype. (**c**) Frequency of γδT cells expressing the indicated markers; each dot represents a mouse. (**d**) IFN-γ and IL-17A expression by hepatic γδT cells at the indicated B6 mouse age over time (*n*=5/time point). (**e**) The host origin (CD45.1^+^ or CD45.2^+^) of hepatic γδT (CD3^+^TCRγδ^+^) and conventional T (CD3^+^TCRγδ^−^ NK1.1^−^) cells was identified by FACS analysis in each mouse of CD45.1/CD45.2 parabiotic B6 mouse pairs at 14 days post surgery (*n*=5 pairs). The data are representative of three independent experiments The mean±s.e.m. is shown. (*P<0.05; **P<0.01; ***P<0.001. one-way ANOVA with post hoc test). IEL, intraepithelial lymphocyte; iLN, inguinal lymph node; mLN, mesenteric lymph node; PC, peritoneal cavity; si, small intestine.

Compared with other immune organs and tissues, hepatic γδT cells predominantly produced high levels of IL-17A, similar to γδT cells from the peritoneal cavity (PC) and lung and significantly higher than those from inguinal lymph nodes (iLNs), the spleen, the thymus, small intestine intraepithelial lymphocytes (IEL), colon IEL and mesenteric LN (mLN) (Fig. 1a,c). In terms of phenotype, hepatic γδT cells exhibited mixed Vγ chain usage, which was also distinct from γδT cells of other organs (Fig. 1b). They were in a more active and mature state, as indicated by higher percentages of CD44^high^CD62L^−^ cells and lower CD24 expression (Fig. 1c). Corresponding with their high IL-17A expression levels, hepatic γδT cells expressed low levels of CD27 (Fig. 1c), which is a fate determinant of γδT cells to express IFN-γ (γδT-1) but not IL-17A (γδT-17)^17^. However, unlike γδT cells of the PC and lung, hepatic γδT cells rarely expressed cytokine receptors including CD121, CD25 and CD127 (Fig. 1c). Interestingly, neonatal mice had low levels of γδT-17 but high levels of γδT-1 cells in the liver (Fig. 1d). As the mice aged, the hepatic γδT-17 cell frequency increased, while that of γδT-1 cells decreased, suggesting that hepatic γδT-17 cells might be induced after birth (Fig. 1d). Overall, hepatic γδT cells exhibited a unique composition and phenotype, indicating that they represent a distinct γδT-cell subtype.

We further characterized the trafficking and homing tendencies of hepatic γδT cells. GFP^+^ splenic and CD45.1^+^ hepatic lymphocytes were transferred separately or together into *Cd45.2*^*+*^ *Tcrd*^*−/−*^ mice (Supplementary Fig. 1a). One day after transfer, splenic γδ T cells travelled randomly into both the spleens and the livers of recipient mice. In contrast, hepatic γδT cells selectively homed only to the liver, and co-transfer with splenic lymphocytes did not change this homing tendency. We confirmed this result by transferring purified hepatic γδT cells into *Rag1*^*−/−*^ mice and found that hepatic γδT cells, particularly γδT-17 cells, homed back to the liver but not to other organs (Supplementary Fig. 1b). To further explore whether hepatic γδT cells reside in the liver, CD45-congenic mice were surgically joined by parabiosis and evaluated for chimerism in various cell populations 2 weeks later. While conventional T cells exhibited substantial chimerism (Fig. 1e), the livers of CD45.1 parabiont mice contained almost all CD45.1^+^ γδT cells with very few, if any, CD45.2^+^ γδT cells; vice versa was observed in CD45.2 parabiont mice (Fig. 1e). Interestingly, PC and thymic γδT cells were also locally retained, but lung, spleen and iLN γδT cells were mutually exchanged (Supplementary Fig. 1c).

Compared with circulating γδT cells, most of the chemokine receptors and integrin molecules were expressed at lower levels on hepatic γδT cells, but C-X-C chemokine receptor 6 (CXCR6) was expressed at extremely high levels on hepatic γδT cells (Supplementary Fig. 2a). However, it was not involved in the liver homing of hepatic γδT cells because the numbers of hepatic γδT cells and γδT-17 cells were not changed in *Cxcr6*^−*/*−^ mice (Supplementary Fig. 2b), although CXCR6 was important for the liver homing of natural killer (NK) and NKT cells^18^,^19^. Moreover, the liver residency of hepatic γδT cells was absent in *Il17a*^−*/*−^ mice. WT and *Il17a*^−*/*−^ mice were surgically joined by parabiosis; 2 weeks later, the WT mouse liver contained few *Il17a*^−*/*−^ mouse-derived γδT cells, but the *Il17a*^−*/*−^ mouse liver contained nearly 50% γδT cells derived from the WT mouse (Supplementary Fig. 2c). These results suggest that γδT cell liver homing might be related to their IL-17 expression ability in unknown mechanism.

In summary, these data collectively indicate that liver-resident γδT cells are indeed a unique γδT-cell subset with low expression levels of cytokine receptors, and that predominantly produce high levels of IL-17A and are retained in the liver.

### The microbiota maintain hepatic γδT-17 cell homeostasis

**Figure 2:**
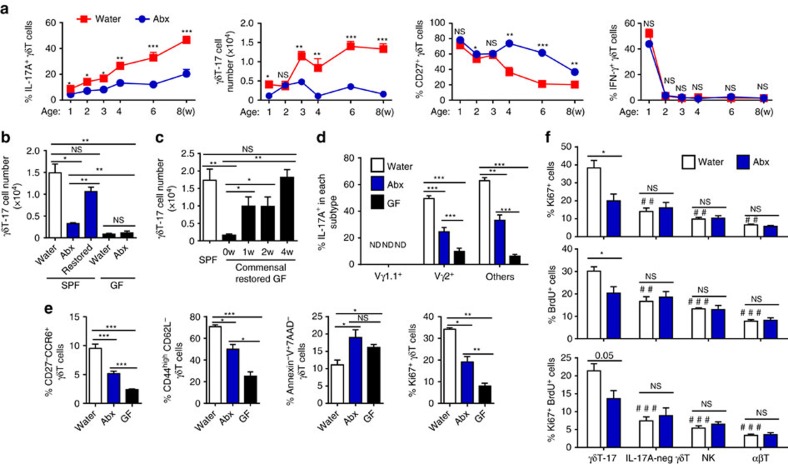
The microbiota maintain the pool of hepatic γδT-17 cells in steady state. (**a**) FACS analysis of IL-17A, CD27 and IFN-γ expression by hepatic γδT cells at the indicated B6 mouse ages over time. The mice began treatment with water alone (Water) or containing antibiotics (Abx) *in utero* through the pregnant mother (*n*=7/time point). (**b**) Five-week-old adult SPF B6 mice and GF mice were fed water alone (Water) or containing antibiotics (Abx) for 4 weeks. Abx-pretreated SPF mice were co-housed with normal mice for an additional 4 weeks to reconstitute their gut bacteria (Restored), and the hepatic γδT-17 cell numbers were evaluated by FACS (*n*=4 for SPF mice, *n*=5 for GF mice). (**c**) Ten-week-old GF mice were co-housed with SPF mice to restore their commensal microbes, and the hepatic γδT-cell number was detected at the indicated time points post co-housing (*n*=9, 4, 5, 5, 10; from left to right). The frequency of IL-17A expression by each hepatic γδT-cell subtype (*n*=10, 7, 5; from top to bottom) (**d**) and the frequency of hepatic γδT cells with the phenotype CD44^high^CD62L^−^, CD27^−^CCR6^+^, Annexin-V^+^7AAD^−^ and Ki67^+^ (*n*=5 per group) (**e**) from Water-treated mice, Abx-treated mice and GF mice were analysed by FACS. (**f**) Control and Abx-treated mice were i.p. injected with 1 mg BrdU three times at 2-day intervals, and BrdU incorporation and Ki67 expression by hepatic γδT-17 (CD3^+^γδTCR^+^IL-17^+^), IL-17A negative γδT (CD3^+^γδTCR^+^IL-17^−^), NK (CD3^–^NK1.1^+^) and αβT (CD3^+^TCRβ^+^) cells were evaluated by FACS. Differences between γδT-17 cells and other cells from control mice (indicated as ‘#’) and differences between Water- and Abx-treated mice of each subtype of cells (indicated as ‘*’) are shown (*n*=6, 4; Water, Abx). The data are representative of more than three independent experiments. The mean±s.e.m. is shown (*P<0.05; **,^##^*P*<0.01; ***,^###^*P*<0.001 one-way ANOVA with post hoc test.).

To explore if the microbiota might influence liver-resident γδT-17 cells, antibiotics were used to clear mouse gut bacteria as previously reported^20^. The antibiotic (Abx)-treated mice had a sharp decrease in commensal microbes, as indicated by the 99% reduction in cultivable bacteria, 95% reduction in bacterial DNA and elongated caecum (Supplementary Fig. 3a–c). Moreover, Abx-treated mice displayed a normal peripheral immune response (Supplementary Fig. 3d). Although the total number and frequency of hepatic B, T, NK, NKT and regulatory T (Treg) cells were normal in Abx-treated mice (Supplementary Fig. 3e,f), the accumulation of liver-resident γδT-17 cells during ontogeny was blocked in mice treated with antibiotics beginning *in utero* through the pregnant mother (Fig. 2a). The reduction in CD27 expression on hepatic γδT cells was also arrested, but the decline of hepatic γδT-1 cells was not affected (Fig. 2a).

To directly observe the interaction between hepatic γδT-17 cells and the microbiota, germ-free (GF) mice were used, which had even more significantly decreased numbers of hepatic γδT-17 cells than Abx-treated SPF mice (Fig. 2b). In addition, treating GF mice with antibiotics did not further reduce their hepatic γδT-17 cell numbers, suggesting that antibiotics were not directly toxic to γδT-17 cells (Fig. 2b). Reconstituting Abx-treated mice with gut commensal microbes was sufficient to restore their reduced hepatic γδT-17 cell numbers to a normal level (Fig. 2b). The kinetics of hepatic γδT-17 cell recovery in commensal microbe-reconstructed GF mice were also studied. Commensal microbe reconstitution recovered the hepatic γδT-17 cell number gradually, reaching a similar level to that in SPF mice after 4 weeks of reconstruction (Fig. 2c). These results indicate that the accumulation of hepatic γδT cells relies on microbiotas.

The reduced hepatic γδT-17 cells in Abx-treated mice and GF mice were not restricted by Vγ chain usage (Fig. 2d). Consistent with the reduced IL-17A expression by γδT cells from Abx-treated mice and GF mice, they were less differentiated and less activated, as indicated by the lower proportions of CD27^–^CCR6^+^ and CD44^high^CD62L^–^ cells (Fig. 2e). Moreover, enhanced apoptosis and reduced proliferation of hepatic γδT cells were also observed after Abx treatment (Fig. 2e). To better understand the specific influence of the microbiota on hepatic lymphocytes, an *in vivo* BrdU incorporation method was used to assay the cell proliferation. Only γδT-17 cells, but not NK, αβT or even IL-17A-negative γδT cells, displayed reduced BrdU incorporation and Ki67 expression in Abx-treated mice, indicating that the microbiota specifically promoted the proliferation of hepatic γδT-17 cells, but not all lymphocytes, in the liver (Fig. 2f). Together, these data indicated that the microbiota sustain liver-resident γδT-17 cell homeostasis by maintaining their normal activation, survival and proliferation.

### Global commensal load affects hepatic γδT-17 cell number

**Figure 3:**
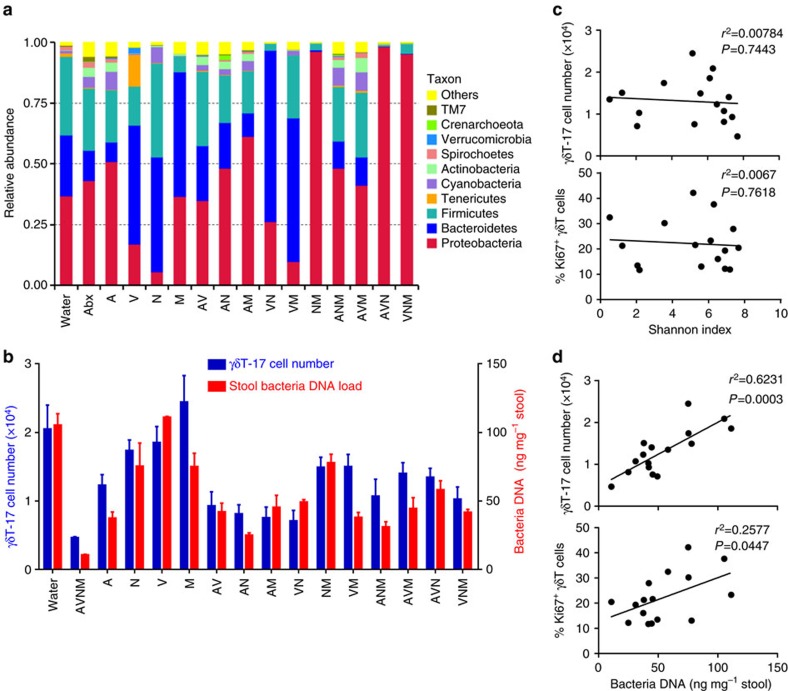
Commensal microbe load is positively correlated with hepatic γδT-17 cell numbers. Mice were fed for 4 weeks with water alone (Water) or containing the following antibiotics for 4 weeks (A=ampicillin; V=vancomycin; N=neomycin; M=metronidazole): all four (AVNM), each one alone or in various combinations (AV, AN, AM, VN, VM, NM, AVN, AVM, VNM and ANM). (**a**) Genomic bacterial DNA was isolated from faecal samples, and the V5–V6 hypervariable region of 16S rDNA was amplified and sequenced. The average percentages of reads for the three experimental groups are shown. (**b**) The left axis indicates the number of hepatic γδT-17 cells determined by FACS, and the right axis indicates the DNA load of bacteria in the faeces. (**c**) The Shannon index of diversity relative to species was calculated, and Pearson correlation curves with the γδT-17 cell number and Ki67 expression are shown. (**d**) The Pearson correlation curves between the γδT-17 cell number and Ki67 expression with the bacterial DNA load. The data are representative of more than three independent experiments (*n*=7 mice per group). Either a representative plot or the mean±s.e.m. is shown.

To screen for bacteria that influence liver-resident γδT-17 cell numbers, different combinations of antibiotics (A, ampicillin; V, vancomycin; N, neomycin; M, metronidazole) were used to treat mice. Different antibiotic mixtures had different targets (Supplementary Fig. 4a,b); thus, we produced a series of mice with commensal microbes at various clusters (Supplementary Fig. 4c) and diversities (Supplementary Fig. 4d). However, although different compositions of microbes were induced in the mice (Fig. 3a), there was almost no correlation between the γδT-17 cell number and the antibiotic type (Fig. 3b) or the bacteria species diversity (Fig. 3c).

Interestingly, the mice with low levels of microbes always had small numbers of hepatic γδT-17 cells, and the mice with high levels of microbes always had large numbers of hepatic γδT-17 cells (Fig. 3b). Indeed, both the numbers and the proliferation percentages of hepatic γδT-17 cells positively correlated with the global microbe DNA loads (Fig. 3d). These results suggest that the global bacterial load is a key factor in the homeostasis of liver-resident γδT-17 cells.

**Figure 4:**
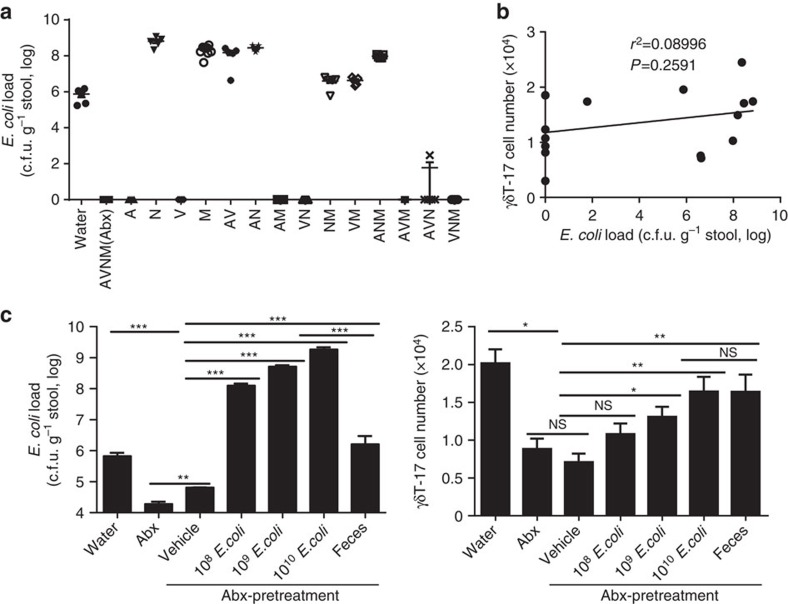
*E. coli*is sufficient but not essential to promote hepatic γδT-17 cells. Mice were treated with antibiotics as described in Fig. 3. (**a**) *E. coli* in fresh stool were plated and counted on an EMB plate (each dot represents a mouse). (**b**) The Pearson correlation curve between the γδT-17 cell number and the *E. coli* load. (**c**) Abx-pretreated mice continued on Abx (Abx) or untreated (Abx-pretreatment) water, and then the Abx-pretreated mice were intragastrically administered vehicle, 10^8^ c.f.u. *E. coli*, 10^9^ c.f.u. *E. coli*, 10^10^ c.f.u. *E. coli* or 10 mg fresh faeces (Faeces). *E. coli* and faeces were from normal mice. The stool *E. coli* load (3 days post transfer) and hepatic γδT-17 cell numbers (3 weeks post transfer) were detected (*n*=5, 6, 6, 6, 8, 8, 7; from left to right). The data are representative of three independent experiments. The mean±s.e.m. is shown (*P<0.05; **P<0.01 one-way ANOVA with post hoc test).

*E. coli,* a typical type of commensal microbe, was chosen to further explore this hypothesis. *E. coli* was completely deleted in eight groups of mice, including the A, V, AM, VN, AVM, AVN, VNM and AVNM groups (Fig. 4a), but some mice without *E. coli* (V, AVM and AVN groups) still had a comparable level of hepatic γδT-17 cells as normal mice (Fig. 3b), suggesting that *E. coli* is not an irreplaceable bacterium for liver-resident γδT-17 cell homeostasis; indeed, there was no correlation with γδT-17 cell numbers (Fig. 4b). However, similar to the transfer of fresh faeces, transferring *E. coli* alone recovered the decline in hepatic γδT-17 cells in Abx-treated mice, with dose dependency in single *E. coli-*transferred mice (Fig. 4c). Thus, these results indicate that the global bacterial load, regardless of species specificity, is important for hepatic γδT-17 cell homeostasis.

### CD1d presents commensal lipid antigen to hepatic γδT-17 cell

To investigate the mechanism by which the microbiota/*E. coli* promote liver-resident γδT-17 cell accumulation, microbiota-derived PAMPs were evaluated. Though TLR agonists, such as poly(I:C) (a TLR3 ligand), could increase the hepatic γδT-17 cell number in WT mice (Supplementary Fig. 5a), administration of Pam3Csk4 (a TLR1/2 ligand), LPS (a TLR4 ligand), CpG (a TLR9 ligand), curdlan (a Dectin-1 ligand) or poly(I:C) could not restore hepatic γδT-17 cells in Abx-treated mice (Supplementary Fig. 5b,c). Moreover, TLR2/TLR4/TLR9 deficiency did not influence hepatic γδT-17 cells (Supplementary Fig. 5d). The second microbiota-associated candidate for regulating hepatic γδT-17 cells was cytokine signalling. However, the levels of liver cytokines (Supplementary Fig. 5e) and the cytokine receptor expressions on hepatic γδT cells (Supplementary Fig. 5f) were unchanged in Abx-treated mice, though CD121 expression on PC γδT cells was reduced (Supplementary Fig. 5f), as previously reported^21^. Neutralizing antibodies against IL-1β and IL-23 *in vivo* reduced the number of PC γδT-17 cells, but not hepatic γδT-17 cells (Supplementary Fig. 5g). Thus, PAMPs and cytokine signals do not support the homeostasis of liver-resident γδT-17 cells.

**Figure 5:**
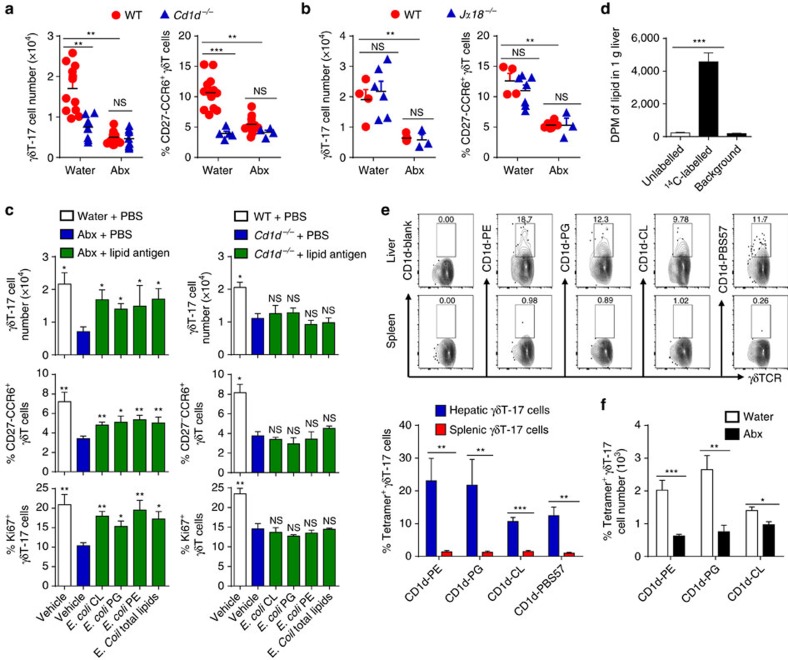
*E. coli*lipid antigens promote hepatic γδT-17 cells in a CD1d-dependent manner. (**a**) Age-matched (3 weeks old) and co-housed WT and *Cd1d*^*−/−*^ mice were fed water alone (Water) or containing antibiotics (Abx) for 4 weeks, and IL-17A expression and phenotype markers of hepatic γδT cells were evaluated by FACS at 7 weeks old (each dot represents a mouse). (**b**) WT and *Jα18*^−*/*−^ mice were co-housed as in **a**, and the IL-17A expression and phenotype markers of hepatic γδT cells were then evaluated by FACS (each dot represents a mouse). (**c**) Abx-treated WT mice or normal water-treated *Cd1d*^*−/−*^ mice were i.p. injected with 20 μg *E. coli* cardiolipin (CL), *E. coli* phosphatidylglycerol (PG), *E. coli* phosphatidylethanolamine (PE) or 50 μg total *E. coli* polar lipid extract six times at 2-day intervals, and IL-17A expression, Ki67 expression and phenotype markers of hepatic γδT cells were evaluated by FACS (*n*=5 per group). (**d**) Abx-treated mice were intragastrically administered 10^10^ c.f.u. of unlabelled or ^14^C-labelled *E. coli*, and the radioactivity of the lipids extracted from the liver was evaluated 6 h later (*n*=3, 4, n/a; from left to right) (**e**) γδT-17 cells (gated CD45^+^CD3^+^TCRγδ^+^IL-17A^+^) from the spleens and livers of WT mice were stained with CD1d tetramer loaded with the indicated lipid antigens, and the representative graph and cumulative data are shown (*n*=4 per group). (**f**) Mice were treated with antibiotics, and the CD1d-lipid antigen tetramer-specific hepatic γδT-17 cell numbers were evaluated by FACS (*n*=4 per group). The data are representative of three independent experiments and shown as the mean±s.e.m. (*P<0.05; **P<0.01; ***P<0.001 one-way ANOVA with post hoc test.).

The liver is rich in lipid antigens and CD1d; thus, we speculated that CD1d/lipid antigen complexes interact with liver-resident γδT-17 cells in the liver. WT and *Cd1d*^*−/−*^ mice were co-housed to share the same microbiota, but *Cd1d*^−*/*−^ mice had a decreased number of hepatic γδT-17 cells with lower proportions of CD27^−^CCR6^+^ cells compared with WT mice, similar to the result observed in Abx-treated WT mice (Fig. 5a). Co-housed NKT-deficient *Ja18*^*−/−*^ mice had similar hepatic γδT-17 cell numbers to WT mice, demonstrating that CD1d sustained hepatic γδT-17 cells directly and independently of NKT cells (Fig. 5b). Moreover, Abx-treatment did not further downregulate the number and frequency of hepatic γδT-17 cells in *Cd1d*^*−/−*^ mice (Fig. 5a). Collectively, these data indicate that the microbiota maintain the homeostasis of liver-resident γδT-17 cells mainly through CD1d signalling.

To explore whether CD1d-associated lipid antigens indeed have a role in this process, Abx-treated WT mice were injected with previously reported CD1d-presenting bacterial lipid antigens, including *E. coli* cardiolipin (CL), PG, PE and total polar lipid extract from *E. coli.* After receiving exogenous *E. coli-*derived lipids, the liver-resident γδT-17 cell number, proliferation and CD27^−^CCR6^+^ expression levels partially but markedly recovered in Abx-treated WT mice but not in *Cd1d*^*−/−*^ mice (Fig. 5c), indicating that lipid antigens act in a CD1d-dependent manner. Moreover, lipid antigens still induced the recovery of the hepatic γδT-17 cell numbers in Abx-treated IL-1/IL-23-neutralized mice (Supplementary Fig. 5h) and TLR2/TLR4/TLR9-deficient mice (Supplementary Fig. 5i), which further suggested that lipid antigens maintain the pool of liver-resident γδT-17 cells in a TLR/cytokine signal-independent manner.

Some microbiota metabolic molecules may reach the liver via the portal vein^22^. To explore if microbiota lipid antigens could reach the liver, ^14^C-glucose was used to label *E. coli* that was then intragastrically delivered into mice. After 6 h, there was high radioactivity in the lipids extracted from the livers of mice transferred with ^14^C-glucose labelled *E. coli* but not in the mice transferred with unlabelled *E. coli* (Fig. 5d). This suggest that microbial lipids can enter the liver at steady state, which allows them to encounter hepatic γδT-17 cells in the liver. γδT-17 cells from both the liver and the spleen were stained with CD1d tetramers loaded with different lipid antigens (PE, PG, CL and PBS57), and liver, but not spleen, γδT-17 cells specifically recognized *E. coli*-derived lipid antigens (PE, PG and CL) and a model antigen PBS57 (ref. 23; Fig. 5e). In addition, these CD1d tetramer-positive γδT-17 cells in the liver decreased markedly after microbiota depletion (Fig. 5f). These results indicate that liver γδT-17 cells specifically recognize microbiota-derived lipid antigens.

**Figure 6:**
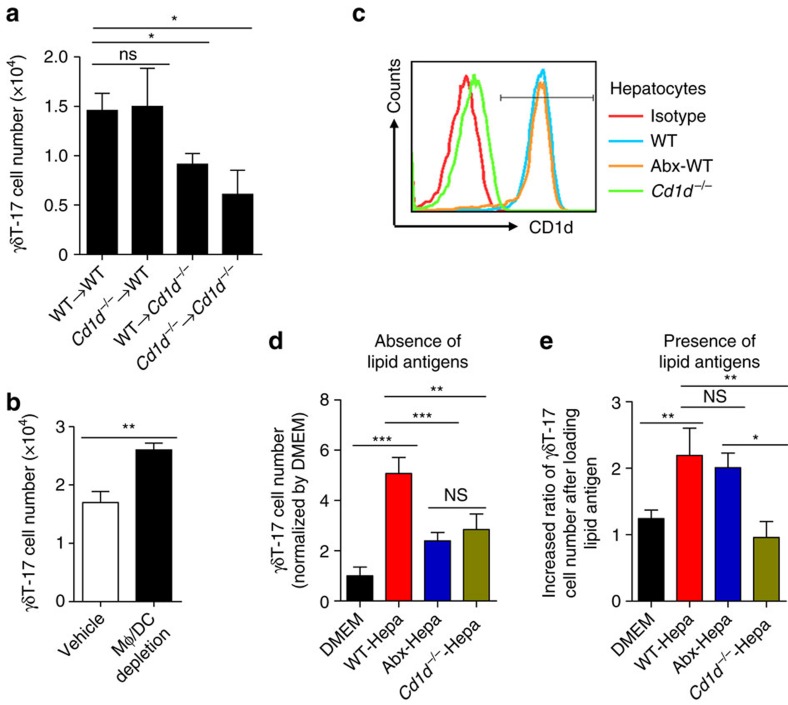
Hepatocytes loaded with lipid antigen promote hepatic γδT-17 cells *in vitro*. (**a**) Quantification of hepatic γδT-17 cells from chimeric mice reconstructed with fetal liver MNCs (*n*=4 per group). (**b**) The numbers of hepatic γδT-17 cells from vehicle- or clodronate liposome-treated WT mice (*n*=4, 5). (**c**) CD1d expression on the indicated hepatocytes. (**d**) Purified hepatic γδT cells from WT mice were co-cultured with the indicated hepatocytes for 3 days, and the γδT-17 cell number (normalized by the DMEM group) was analysed by FACS (*n*=6 wells per group). (**e**) Purified γδT cells were co-cultured with lipid antigen-loaded hepatocytes for 3 days. The increased ratios of γδT-17 cell numbers compared with those without added lipid antigens are shown (*n*=6 wells per group). The data are representative of three independent experiments and shown by the mean±s.e.m. (*P<0.05; **P<0.01; ***P<0.001 unpaired Student's *t*-test (**b**), one-way ANOVA with post hoc test (**a**,**d**,**e**).

Next, we asked which cells expressed CD1d stimulate hepatic γδT-17 cells. Using fetal liver chimeric mice, we found that the number of hepatic γδT-17 cells decreased only when CD1d was deficient in non-hematopoietic cells, but not in hematopoietic cells (Fig. 6a). Indeed, when using clodronate to deplete macrophages and dendritic cells (DCs), both of which are major hematopoietic cells expressing CD1d, the number of hepatic γδT-17 cells was not reduced but rather slightly increased (Fig. 6b). Interestingly, the hepatocytes expressed a high level of CD1d (Fig. 6c), as previously reported^24^. In the *in vitro* co-culture system, WT hepatocytes displayed a higher ability to promote γδT-17 cells than hepatocytes from Abx-treated mice and *Cd1d*^*−/−*^ mice (Fig. 6d); this difference might arise from the reduced number of endogenous lipid antigens presented by CD1d on Abx-treated hepatocytes. After supplying exogenous lipid antigens, WT or Abx-treated, but not *Cd1d*^−*/*−^, hepatocytes induced an approximately twofold increase in the γδT-17 cell number compared with those without exogenous lipid antigens added (Fig. 6e). These results indicate that hepatocytes can directly present commensal/*E. coli* lipid antigens through CD1d to promote liver-resident γδT-17 cell expansion.

### Hepatic γδT-17 cell accelerate HFD-induced NAFLD progression

Using an *Il17ra*^−*/*−^ mouse, Harley *et al*.^25^ showed that IL-17A signalling could accelerate NAFLD through recruiting neutrophils and inducing nicotinamide adenine dinucleotide phosphate (NADPH) oxidase-dependent ROS, which ultimately induced hepatocellular damage. We wondered if the predominant liver-resident γδT-17 cell subtype was the source of IL-17A during NAFLD, and, importantly, whether the microbiota promoted NAFLD through these cells.

**Figure 7:**
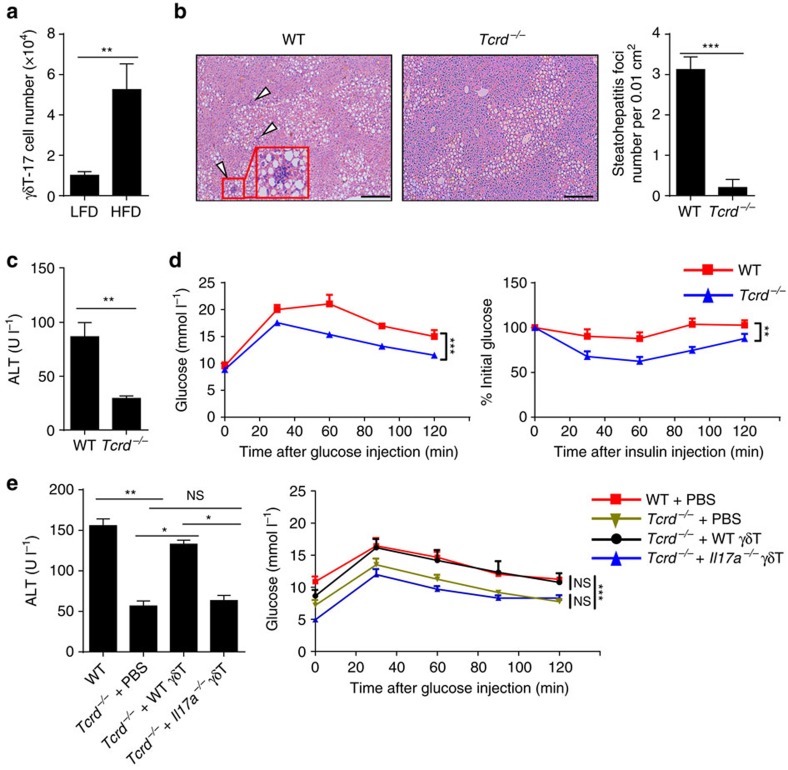
Hepatic γδT-17 cells are essential for inducing NAFLD. (**a**) WT mice were placed on an HFD for 24 weeks, and the hepatic γδT-17 cell number was detected by FACS (*n*=4, 5). (**b**–**d**) WT mice and *Tcrd*^−*/*−^ mice were placed on an HFD for 24 weeks. (**b**) Representative liver histology (H&E staining, scale bar, 200 μm); arrowheads indicate the steatohepatitis foci of inflammation with clusters of inflammatory cells, and the red rectangle indicates a 4 × zoom of the gated region (*n*=4, 5). (**c**) The serum ALT level (*n*=4, 5), (**d**) glucose tolerance test (GTT) curve and insulin tolerance test (ITT) curve were assessed (*n*=4, 9). (**e**) HFHCD-fed *Tcrd*^−*/*−^ mice were either i.v. transferred with WT hepatic γδT cells or *Il17a*^−*/*−^ hepatic γδT cells (2 × 10^4^, once per week) from the 4th week to the 10th week during HFHCD treatment, and the serum ALT level and GTT curve were evaluated (*n*=4 per group). The data are representative of three independent experiments and shown by the mean±s.e.m. (*P<0.05; **P<0.01; ***P<0.001 unpaired Student's *t*-test (**a**, **b**, **c**), one-way ANOVA post hoc test (**e**, left panel), two-way ANOVA test (**d**, **e** right panel).

High-fat diet (HFD)-fed mice displayed elevated numbers of γδT-17 cells in several organs, including the liver (Fig. 7a, Supplementary Fig. 6a), suggesting that hepatic γδT-17 cells might be one of the main sources of IL-17A in the liver during NAFLD. Though *Tcrd*^−*/*−^ mice a body weight increase comparable to that of WT mice (Supplementary Fig. 6b), they were protected from NAFLD by displaying reduced steatohepatitis, reduced liver damage and a catabatic glucose dysmetabolism (Fig. 7b–d). The reduced NAFLD symptoms in *Tcrd*^−*/*−^ mice were also displayed in another mouse NAFLD model induced by high-fat/high-carbohydrate diet (HFHCD) (Fig. 7e), and transfer of HFHCD-fed mice with WT hepatic γδT cells, but not with *Il17a*^−*/*−^ hepatic γδT cells, accelerated NAFLD in *Tcrd*^−*/*−^ mice (Fig. 7e). These results demonstrate that hepatic γδT-17 cells can accelerate NAFLD.

**Figure 8:**
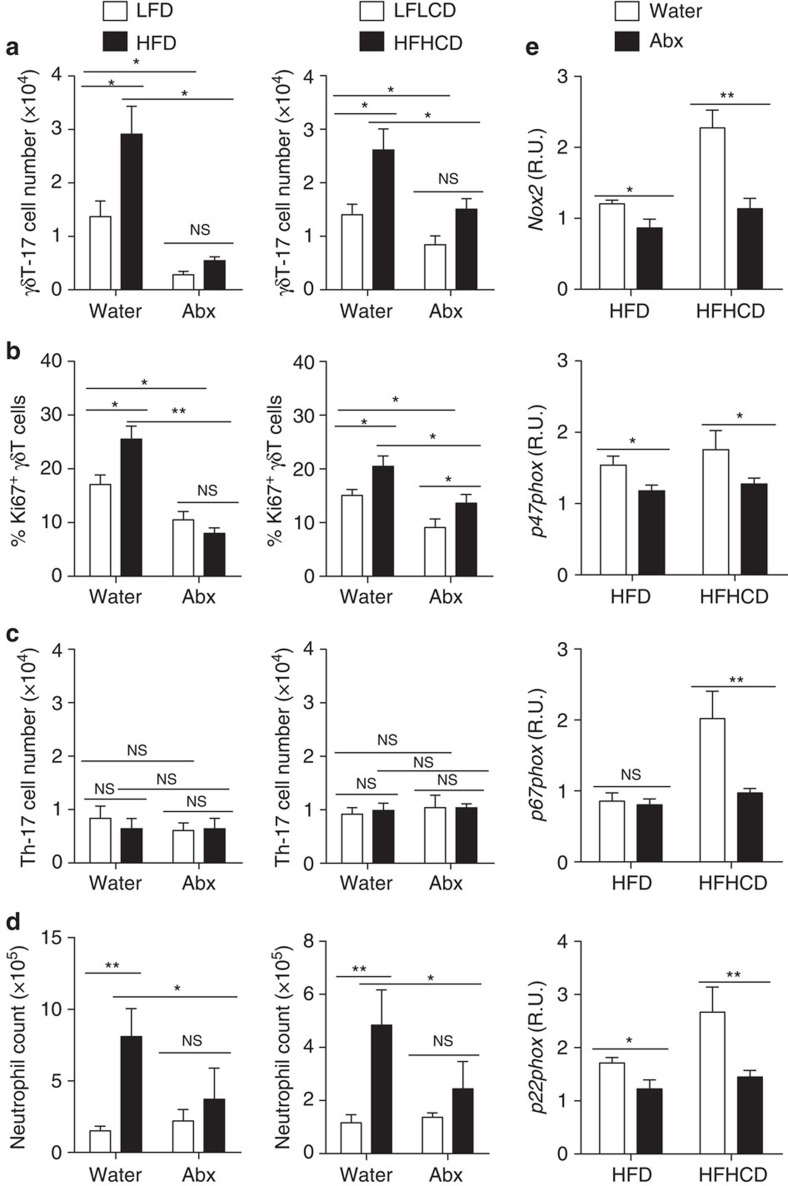
The microbiota promote the expansion of liver-resident γδT-17 cells during HFD/HFHCD-induced NAFLD. Normal water-fed mice and Abx-treated B6 mice were placed on an HFD (LFD as control) and an HFHCD (LFLCD as control) for 24 weeks. (**a**) The hepatic γδT-17 cell number, (**b**) Ki67 expression of hepatic γδT cells, (**c**) hepatic Th-17 cell number and (**d**) neutrophil number in the liver were detected by FACS. (**e**) Water- and Abx-treated B6 mice were placed on an HFD or an HFHCD for 12 weeks. The hepatic mRNA expression levels of *Nox2*, *p47phox*, *p67phox* and *p22phox* were evaluated. The data are representative of three independent experiments with five mice per group and shown by the mean±s.e.m. (*P<0.05; **P<0.01; ***P<0.001 one-way ANOVA with post hoc test.).

Next, we asked whether the microbiota were involved in hepatic γδT-cell expansion during NAFLD. Both HFD and HFHCD triggered the increase and proliferation of hepatic γδT-17 cells (Fig. 8a,b). However, after depleting commensal microbes, Abx-treated mice that started with lower hepatic γδT-cell numbers had only slight increases compared with the baseline of control mice, and the numbers was far lower than the levels observed in HFD/HFHCD-induced NAFLD mice (Fig. 8a,b). Another candidate for microbiota associated-IL-17A production was Th-17 cells; unlike Th-17 cells in the intestine^26^ and skin^27^, which can be induced to proliferation by their local microbiota, the numbers of hepatic Th-17 cells did not decrease in Abx-treated mice, and HFD and HFHCD did not trigger increased hepatic Th-17 cells, indicating the irrelevant role of hepatic Th-17 cells in this process (Fig. 8c). The downstream effectors of IL-17A during NAFLD^25^, the numbers of neutrophils (Fig. 8d), and the mRNA levels of NADPH oxidase enzymes (Fig. 8e) also decreased in the livers of Abx-treated mice.

**Figure 9:**
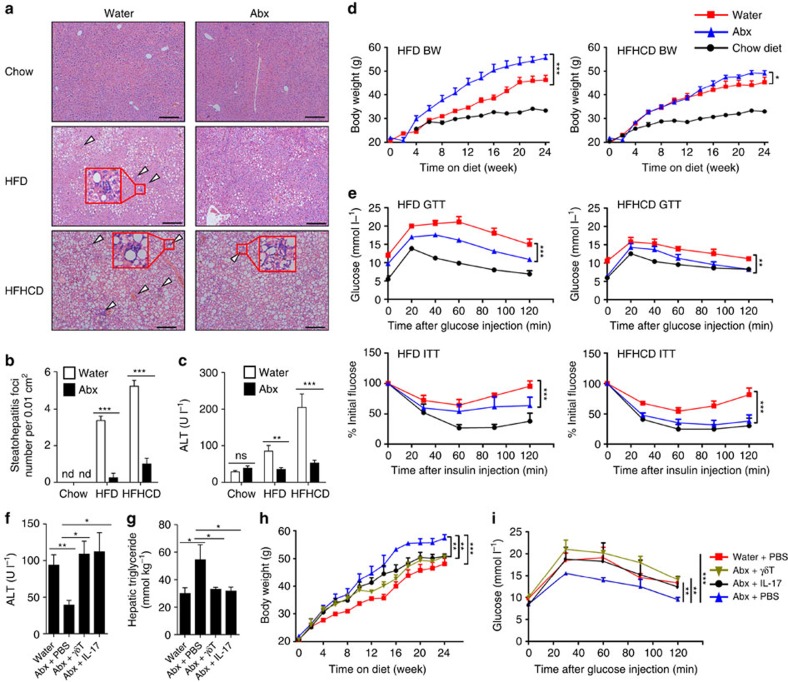
The microbiota accelerate HFD/HFHCD-induced NAFLD through hepatic γδT-17 cells. (**a**–**e**) Mice were treated as described in Fig. 8. (**a**) Representative liver histology (H&E staining, scale bar, 200 μm); arrowheads indicate the steatohepatitis foci of inflammation with clusters of inflammatory cells, and the red rectangle indicates a 4 × zoom of the gated region. (**b**) Numbers of steatohepatitis foci counted in **a** (*n*=5 per group). (**c**) Serum ALT level, (**d**) body weight curve and (**e**) glucose tolerance test (GTT) and insulin tolerance test (ITT) curves are shown (*n*=5 per group). (**f**–**i**) HFD-fed, Abx-treated mice were either injected with IL-17A (500 ng, once per week) or transferred with hepatic γδT cells (2 × 10^4^, once per 2 weeks) from the 4th week to the 24th week of HFD treatment, (**f**) The serum ALT level, (**g**) hepatic triglyceride level, (**h**) body weight curve and (**i**) GTT curve are shown (*n*=5 per group). The data are representative of three independent experiments and shown by the mean±s.e.m. (*P<0.05; **P<0.01; ***P<0.001 one-way ANOVA with post hoc test (**b**,**c**,**f**,**g**), two-way ANOVA test (**d**,**e**,**h**,**i**).

More importantly, Abx-treated mice exhibited a similar alleviation of NAFLD as *Tcrd*^−*/*−^ mice fed with HFD/HFHCD, as indicated by the reduced steatohepatitis (Fig. 9a,b), the reduced ALT level (Fig. 9c), the elevated body weight (Fig. 9d) and the catabatic glucose dysmetabolism (Fig. 9e). The reduced liver damage, elevated hepatic triglyceride level, elevated body weight and catabatic glucose dysmetabolism in Abx-treated mice could be reversed by transferring γδT cells or IL-17A protein (Fig. 9f–i). Together, these data suggest that the microbiota function as a co-factor to accelerate HFD/HFHCD-triggered NAFLD *via* increasing hepatic γδT-17 cells.

## Discussion

To our knowledge, our study is the first to describe the unique characteristics and mechanisms of liver-resident γδT cells controlled by the microbiota. First, they are liver resident without exchanging with circulating γδT cells (Fig. 1e and Supplementary Fig. 1a–c); second, hepatic γδT cells predominantly produce a high level of IL-17A (Fig. 1a–d); third, they specifically recognize (Fig. 5e) and are promoted by (Figs 5c and 6d,e) CD1d/commensal lipid antigen.

γδT-17 cells originate from fetal γδ thymocytes and are preferentially located in barrier tissues^28^. The heterogeneity of γδT cells in different locals is more common than previously thought. Skin γδT-17 cells are compartmentally controlled by the skin, but not gut, microbiota^27^; IL-1R signalling has an important role in microbiota-mediated IL-17A production by PC γδT cells^21^; IL-17A production by lung-resident γδT cells requires IL-6 signalling instead^29^; dermal γδT cells preferentially rely on IL-1 and MyD88 signalling to produce IL-17A^30^; the γδT-cell subtype in the colonic lamina propria produces IL-17A in an IL-23-independent manner^31^. Thus these findings together with the mechanism of liver γδT-cell homeostasis found by us, further indicate that the host–microbiota interaction at distinct barrier sites occurs in a tissue-specific manner^32^.

IL-17A production by γδT cells is often elicited by cytokines or TLRs without prior antigen exposure (termed ‘natural γδT-17’ cells)^33^, and we showed that TLR and IL-1/IL-23 signalling was not responsible for liver γδT-17 cell homeostasis in a physiological state (Supplementary Fig. 5). These two seemingly contradictory facts together exposed the mystery between γδT-cell antigen recognition and TLR/cytokine recognition. Increasing numbers of studies have indicated that encountering antigen is a prerequisite for γδT cells to respond to inflammatory cytokines^34^,^35^. Until now, the antigen specificity or antigen requirement of the ‘natural γδT-17’ cells has remained unknown or controversial^10^,^28^. However, although γδT cells in the mucosa and epithelium are well-suited to recognize microbial components, their recognized antigens remain scarce^28^. In our study, we demonstrate for the first time that commensal lipid antigens can reach the liver (Fig. 5d) and be presented by CD1d to the γδTCR of liver-resident γδT cells (also a type of ‘natural γδT-17’) (Figs 5e and 6d,e), which supports the development and homeostasis of this cell subset.

The lipid antigens that specifically link CD1d and the γδTCR include bacterial CL^36^, synthetic α-GalCer^37^, sulfatide^11^,^38^, synthetic phosphatidylcholine, PE, PG^12^ and synthetic and natural PE^39^. Among these, CL, PG and PE can be derived from both bacteria and stressed host cells^40^,^41^. Indeed. *E. coli* CL, PG and PE could partly recover the decreased hepatic γδT-17 cells in Abx-treated mice (Fig. 5c); nevertheless, none of these antigens could completely recover the decline, suggesting that other lipid antigens may also have roles in this process. We showed that liver γδT-17 cells could also be stained by CD-1d-PBS57 tetramer (an improved form of α-GalCer^23^) (Fig. 5e), which may be a result of the cross-reactivity of the CD1d-lipid antigens recognized by the γδTCR. The cross-reactivity of CD1d-phospholipids and CD1d-α-GalCer^37^, as well as that of CD1d-CL and CD1d-phospholipids^42^, were also observed in other γδTCR studies.

Several types of hepatic cells, including endothelial cells, Kupffer cells and DCs, can express CD1d, but the strongest CD1d-expressing cell is hepatocytes, which are also critical lipid metabolic factories^24^. Furthermore, CD1d expression on hepatocytes is the main component of hepatic T-cell-lipid antigen recognition^13^. Investigators previously observed that CD1d on hepatocytes could activate NKT cells^14^,^15^,^16^. Our study demonstrated that hepatocytes can also promote γδT-17 cells in the presence of lipid antigens (Fig. 6c–e). The difference in lipid antigen recognition by the TCR of γδT cells and that of NKT cells is an interesting topic. For example, although CD1d can present α-GalCer to both γδT cells and NKT cells, the mode of γδTCR–CD1d–α-GalCer recognition appears to be markedly different from that of NKT recognition^37^. Thus, the difference between NKT and γδT cells in CD1d recognition of microbiota-derived lipid antigens requires further investigation.

NAFLD is induced by chronic inflammation in obesity, and the progression of NAFLD is the comprehensive result of hepatic immune cells, including Kupffer cells^43^, NK cells^44^ and NKT cells^45^, and liver metabolic cells, including hepatocytes and hepatic stellate cells^44^,^46^. However, although the metabolic relationship between the microbiota and fatty liver has been shown, how the microbiota influences the hepatic immune response during NAFLD remains unclear^47^,^48^,^49^. In this study, we showed that microbiota-maintained liver-resident γδT-17 cells were the main source of IL-17A and could significantly accelerate NAFLD (Fig. 7). An interesting finding was the uncoupling of decreased liver inflammation (Fig. 9a–c) and increased body weight (Fig. 9d) in Abx-treated mice, which was also found by other groups in *Il17ra*^−*/*−^ mice^25^ and Abx-treated mice^50^. However, this uncoupling was not found in *Tcrd*^−*/*−^ mice, who displayed reduced liver inflammation (Fig. 7b–e) but comparable body weights to those of WT mice, as shown by us (Supplementary Fig. 6b) and others^51^. This suggests that different mechanisms are used by IL-17A to promote hepatic inflammation and lipid metabolism and that the lipid metabolism pathway might be compensated for by other signals in *Tcrd*^−*/*−^ mice.

After depleting commensal microbes, hepatic γδT-17 cells did not increase (Fig. 8a), but they did increase in the presence of microbiota during NAFLD. Nevertheless, same as γδT-17 cells in the liver, γδT-17 cells in the gut and other tissues also increase during NAFLD (Supplementary Fig. 6a), mainly because NAFLD is a systemic disease with inflammation in multiple organs^52^. Thus, though there is no mutual exchange or circulation between hepatic γδT cells (Fig. 1e) and gut γδT cells^53^ at steady state, we still cannot fully exclude the possibility that a portion of gut γδT-17 cell would traffic to the liver in the disease state. However, instead of the cellular traffic model, we depict a molecular traffic model by displaying that *E. coli* lipid can reach the liver from gut (Fig. 5d); then the lipid antigen can be presented by hepatocyte-expressed CD1d to directly stimulate hepatic γδT cells (Fig. 6c–e) and ultimately alleviate hepatic γδT-17 cell clones that are specific to CD1d-lipid antigen tetramer (Fig. 5e). Nevertheless, four possible mechanisms exist for the accumulation of hepatic γδT-17 cell during NAFLD from our analysis. First, the excessive accumulation of fat-related lipid antigens during NAFLD may further affect hepatic γδT-17 cells; because it is difficult to distinguish the sources of lipids *in vivo*, there is still more work to do. Second, because NAFLD is always accompanied by the over growth of commensal microbes^47^,^48^,^49^, an elevated global microbe load or several specific bacteria may induce a higher number of hepatic γδT-17 cells as described in our study. Third, HFD-induced inflammation may stimulate the microbiota-maintained hepatic γδT-17 cells as a bystander effect. Forth, there may be a portion of γδT-17 cells activated in other tissues (for example, gut) traffic into the liver.

Similar to that observed in the mouse liver, healthy human livers also contain a distinct Vδ3^+^ γδT-cell subset^54^; interestingly, they recognize CD1d and release IL-17A after activation^55^. However, their detailed characteristics and precise mechanisms are unclear. Using a mouse model, our work demonstrates for the first time that hepatic γδT cells are a unique liver-resident subset. Hepatocyte CD1d could present gut commensal lipid antigens to hepatic γδT cells, which drove them to predominantly produce IL-17A and maintained their haemostasis. Our results reveal a novel crosstalk between metabolism and immunity in the liver–gut axis and also suggest a tissue-specific interaction between the microbiota and immune cells in the liver (Supplementary Fig. 7).

## Methods

### Mice

C57BL/6 mice were purchased from the Shanghai Laboratory Animal Center (SLAC, Chinese Academy of Sciences); *Rag1*^*−/−*^ mice were obtained from the Model Animal Research Center (Nanjing University); *Cxcr6*^*gfp/gfp*^ and CD45.1^+^ mice were purchased from the Jackson Laboratory; *Tcrd*^*−/−*^ mice were a gift from Dr Zhinan Yin (Nankai University); *Tlr2*^*−/−*^, *Tlr4*^*−/−*^ and *Tlr9*^*−/−*^ mice were a gift from Dr Shaobo Su (Sun Yat-sen University); *Il17a*^*−/−*^ mice were a gift from Dr Zhexiong Lian (University of Science and Technology of China (USTC)); and *Jα18*^*−/−*^ and *Cd1d*^*−/−*^ mice were a gift from Dr Li Bai (USTC). All of the above mice were housed in a specific pathogen-free facility and all of the animal protocols were approved by Local Ethics Committee for Animal Care and Use at University of Science and Technology of China. Germ-free mice were purchased from SLAC and housed in the germ-free facility at SLAC according to their animal care regulations. The sample size was determined by the ‘resource equation’ method, taking into account the possible reduction of diet/drink treatment.

### Mouse treatment

Six- to ten-week-old male mice were used in most of the experiments with exceptions, such as the use of neonatal mice in the ontogeny experiment and the use of 30-week-old mice in the HFD/HFHCD-induced NAFLD experiment. Commensal microbes were depleted using antibiotics as previously reported^20^, the detailed method are followed. Four kinds of antibiotics including ampicillin (1 g l^−1^), vancomycin (0.5 g l^−1^), neomycin sulfate (1 g l^−1^) and metronidazole (1 g l^−1^) were dissolved in sterile water and stored in 4 °C no more than a week before using. This antibiotic-contained water was supplied as drinking water to adult mice and pregnant mice for more than 4 weeks and was changed every 3 days. The volume of drinking water and the body weights of the mice were monitored twice a week. Mice exhibiting more than a 30% decline in body weight were removed. After antibiotic treatment stopped, Abx-treated mice were co-housed with normal mice for 4 weeks to reconstitute commensals.

For the PAMP restoration experiment, WT mice and Abx-treated mice were intraperitoneally (i.p.) injected with 50 μg curdlan, 50 μg Pam3csk4, 100 μg LPS, 100 μg poly(I:C) or 50 μg CpG (all from Sigma) and harvested 1 day later. For cytokine neutralization, the mice were intravenously (i.v.) injected with 50 μg control IgG, anti-IL-1β or anti-IL-23 antibody twice at 3-day intervals and harvested 3 days later. For the lipid antigen restoration experiment, the mice were i.p. injected with 20 μg *E. coli* CL, PG or PE or 50 μg *E. coli* polar lipid extract (all from Avanti Polar Lipids, Alabama) six times at 2-day intervals and harvested 2 days after the last injection. For macrophage and DC depletion, the mice were i.v. injected with 200 μl Cl2MDP-Lip (Vrije Universiteit) twice at 3-day intervals and harvested 3 days later. All of the mice receiving the above treatment were randomly assigned to different groups, but the investigators were not blinded to the treatments or genotypes.

### Bacterial diversity analysis

Fresh stool samples were collected and weighed, and bacterial DNA was extracted from the stool using a QIAamp Fast DNA Stool Mini Kit (Qiagen). The 16S rRNA gene was analysed to determine the bacterial composition and diversity using an Illumina MiSeq (Novogene Bioinformatics Technology Co., Ltd).

For bacterial titre analysis, fresh stools were collected and homogenized in sterile PBS. The serially diluted homogenates were plated onto blood agar plates (for total cultivable bacterial determination) or eosin methylene blue (EMB; for *E. coli* determination) plates at 37 °C for 24 h. The colonies were distinguished by their biochemical reactions and counted.

### Faeces/*E. coli* transfer

Mice were treated with antibiotics for 4 weeks, and their antibiotic-containing water was replaced with antibiotic-free water. Then, the mice were intragastrically administered 0.2 ml fresh faeces (50 mg ml^−1^) or 10^8^/10^9^/10^10^ c.f.u. of *E. coli* that was isolated from an EMB plate and expanded in LB. The mice were analysed 3 weeks later.

### ^14^C labelling of *E. coli*

*E. coli* was ^14^C labelled as previously reported^56^ with a slight modification. Briefly, *E. coli* was isolated from fresh mouse faeces using an EMB plate, expanded in M9 minimal medium to OD_600_=0.4 and spiked with 25 μCi/L ^14^C-glucose (NEC042V250UC, PerkinElmer) for 16 h. A total of 10^10^ c.f.u. of unlabelled or ^14^C-labelled *E. coli* was intragastrically delivered to the mice. The livers were collected 6 h later, the lipids in the liver were extracted using a methanol/chloroform (2:1) mixture and radioactivity was assessed.

### Parabiosis

CD45.1^+^ and CD45.2^+^ mice were joined by parabiosis for 2 weeks as previously described^57^. Age matched and co-housed mice were anaesthetic and shaved. An incision along the lateral aspect of each mouse was made. The mice were then sutured together at the elbow and knee as well as at the skin around the incision. Mice were i.p. injected with 5% glucose and 0.9% sodium chloride to recover energy and water. Buprenex was used to relieve pain. Sulfatrim was added to the drinking water for 7 days.

### NAFLD model

Mice were placed on two model diets and matched control diets. HFD (60% of kcal fat, carbohydrate 20% kcal, protein 20% kcal; Research Diets #D12492) and LFD (10% of kcal fat, carbohydrate 70% kcal, protein 20% kcal; Research Diets #D12450B) and the corresponding HFHCD (fat 42% kcal, carbohydrate 42.7% kcal, protein 15.3% kcal; TROPHIC Animal Feed High-Tech Co. #TP26301^+^ carbohydrates (18.9 g l^−1^ sucrose+23.1 g l^−1^ fructose) in drinking water) and LFLCD (fat 12.5% kcal, carbohydrate 68.1% kcal, protein 19.4% kcal; TROPHIC Animal Feed High-Tech Co. #TP26323+carbohydrate-free drinking water). Fresh food was supplied twice a week, and the food and drink consumption were quantified throughout the experiment. The body weights of the mice were monitored weekly.

For the IL-17A and hepatic γδT-cell transfer experiment, Abx-treated mice were i.v. injected with IL-17A protein (PeproTech, 500 ng, once per week) or purified hepatic γδT cells (2 × 10^4^, once per 2 weeks) from the 4th week on an HFD until harvesting at the 24th week. *Tcrd*^−*/*−^ mice were i.v. injected with purified hepatic γδT cells (2 × 10^4^, once per week) from the 4th week on a HFHCD until collecting at the 10th week.

For the GTT test, mice were fasted for 12 h and i.p. injected with 1 g kg^−1^ glucose; for the ITT test, mice were fasted for 3 h and i.p. injected with 0.5 U kg^−1^ human fast-acting insulin (Lilly France). Tail vein blood was collected both pre-injection and at various times post injection of glucose or insulin. The glucose levels in the blood were assayed by an automated glucometer (LifeScan). Serum ALT and AST were determined using an automated Chemray 240 clinical analyzer (Rayto, Shenzhen, China). Liver samples that were fixed in 4% paraformaldehyde and paraffin-embedded were sliced into 5-μm-thick sections and stained with H&E. Lipids in the frozen liver tissue were extracted using a methanol/chloroform (2:1) mixture and dissolved in isopropyl alcohol. The triglyceride level was detected using a kit (Huili, China).

### Cell preparation

Mice were killed, and the iLNs, spleens, livers, lungs, thymi, intestines, colons and mesenteric LNs were collected. Mononuclear cells (MNCs) from each organ were then separated as previously described with slight changes^58^. Briefly, LNs and thymi were passed through a 200-gauge steel mesh and washed with PBS. Spleens were first passed a 200-gauge steel mesh then RBC lysed and washed with PBS. Livers were passed a 200-gauge steel mesh and the cell pellet were collected in the flow through, the MNCs in the pellets were isolated by gradient centrifugation with 40 and 70% Percoll. Lungs were first excised and minced to small pieces, then digested with 0.1% collagenase I for 1 h at 37°, the large pieces of lung were removed by filtering and the MNCs in the flow through were obtained by gradient centrifugation with 40 and 70% Percoll. Intestines and colons were surgically exclude the peyer’s patch and then excised to small pieces, digested with 1 mM DTT for 15 min at 37 °C and passed a 200-gauge mesh, IEL in the flow through were isolated by gradient centrifugation with 40 and 70% Percoll, the unfiltered tissue was further digested with collagenase IV for 1 h at 37 °C and filtered with 200-gauge mesh, LPL in the flow through were isolated by gradient centrifugation with 40 and 70% Percoll. PC MNCs were obtained by peritoneal lavage using cold PBS. Hepatic γδT cells were purified using a mouse TCRγδ^+^ T-Cell Isolation Kit (Miltenyi Biotec).

### Fetal liver MNC chimera

To construct a hematopoietic chimera, 2 × 10^6^ fetal liver MNCs from an E18.5 mouse were transferred to lethally irradiated 10-week-old mice (11 Gy, 1 day before transfer). Hepatic γδT cells were analysed 20 weeks later.

### *In vitro* co-culture

Hepatocytes were separated using a previously described two-step perfusion method^59^. Briefly, mice were anaesthetized and the portal vein was cannulated. The liver was first digested with 0.5 mM EGTA and then digested with 0.05% collagenase IV, the digested liver resuspension was passed a 200-gauge steel mesh and the hepatocytes in the flow through were isolated by 40% Percoll. Hepatocytes (5 × 10^4^ per well) were pre-cultured for 24 h to adhere, during which mixed lipid antigens (2.5 μg ml^−1^ PG, PE and CL, dissolved in ethanol and chloroform) were loaded or not loaded onto the cells. Then, the hepatocytes were washed and co-cultured with purified hepatic γδT cells (1 × 10^4^ per well) for 3 days. IL-17A expression was detected by FACS, and the cell number was counted.

### Flow cytometry analysis

Freshly isolated MNCs were blocked and incubated with the indicated fluorescent mAbs for 30 min at 4 °C. The cells were stimulated with 50 ng ml^−1^ PMA (Sigma) and 1 μg ml^−1^ ionomycin (Sigma) and treated with 10 μg ml^−1^ monensin (Sigma), and the cells were then blocked with rat serum and stained for surface markers, fixed, permeabilized and labelled with the indicated intracellular antibody. Antibody information is summarized in Supplementary Table 1. All samples were collected on an LSRII flow cytometer (BD Biosciences) and analysed using FlowJo software (Tree Star). The gating strategy is showed in Supplementary Fig. 8.

### CD1d-lipid antigen tetramer

Brilliant Violet 421-labelled PBS-57-loaded and unloaded mCD1d tetramers were kindly provided by the NIH tetramer core. Unloaded mCD1d tetramer (1 mg ml^−1^, 100 μl) was conjugated with lipid antigens (1 mg ml^−1^ PE, PG or CL, 20 μl) in PBS buffer containing 1 μM pepstatin, 1 μg ml^−1^ leupeptin and 2 mM EDTA, which was then washed and concentrated using a 30 K microconcentrator (Amicon Ultra-15, Millipore UFC903024). The cells were stained with other antibodies as described in the flow cytometry analysis section, collected on a SP6800 spectral analyzer (Sony Biotechnology Inc.), and analysed using FlowJo software with a gating strategy showed in Supplementary Fig. 8.

### BrdU incorporation

Mice were i.p. injected with 1 mg BrdU three times at 2-day intervals. The BrdU^+^ cell frequency was evaluated according to the FITC BrdU Flow Kit instructions (BD Pharmingen).

### Quantitative RT–PCR

Total RNA from liver tissue was extracted using TRIzol reagent (Invitrogen). Gene expression was analysed according to the instructions of the SYBR Premix Ex Taq kit (Takara) and quantified using the ΔΔCt method. All primers (Supplementary Table 2) were synthesized by Sangon (Shanghai, China).

### Statistics

Student’s *t*-test for two groups and a one-way ANOVA for more than two groups were used to determine statistically significant differences. A two-way ANOVA test was used to determine differences in the GTT and ITT tests. Differences achieving values of *P*<0.05 were considered statistically significant.

### Data availability

The data that support the findings of this study are available within the article and its Supplementary Information files or from the corresponding authors on request.

## Additional information

**How to cite this article:** Li, F. *et al*. The microbiota maintain homeostasis of liver-resident γδT-17 cells in a lipid antigen/CD1d-dependent manner. *Nat. Commun.* **8,** 13839 doi: 10.1038/ncomms13839 (2017).

**Publisher’s note**: Springer Nature remains neutral with regard to jurisdictional claims in published maps and institutional affiliations.

## Supplementary Material

Supplementary Information Supplementary Figures and Supplementary Tables
