# Erratum: The microbiota maintain homeostasis of liver-resident γδT-17 cells in a lipid antigen/CD1d-dependent manner

**DOI:** 10.1038/ncomms15265

**Published:** 2017-04-06

**Authors:** Fenglei Li, Xiaolei Hao, Yongyan Chen, Li Bai, Xiang Gao, Zhexiong Lian, Haiming Wei, Rui Sun, Zhigang Tian

Nature Communications 8 Article number:13839 ; DOI: 10.1038/ncomms13839 (2017); Published 01 09 2017; Updated 04 10 2017

The HTML version of this Article previously published had an incorrect volume number of 7; it should have been 8. This has now been corrected in the HTML; the PDF version of the paper was correct from the time of publication.

