# Supplementary material for: The microbiota maintain homeostasis of liver-resident γδT-17 cells in a lipid antigen/CD1d-dependent manner
Source: Nat Commun. 2017 Jan 9;8:13839. doi: 10.1038/ncomms13839 (PMC5227332; doi:10.1038/ncomms13839)
Supplement: Supplementary Information — Supplementary Figures and Supplementary Tables [file ncomms13839-s1.pdf]

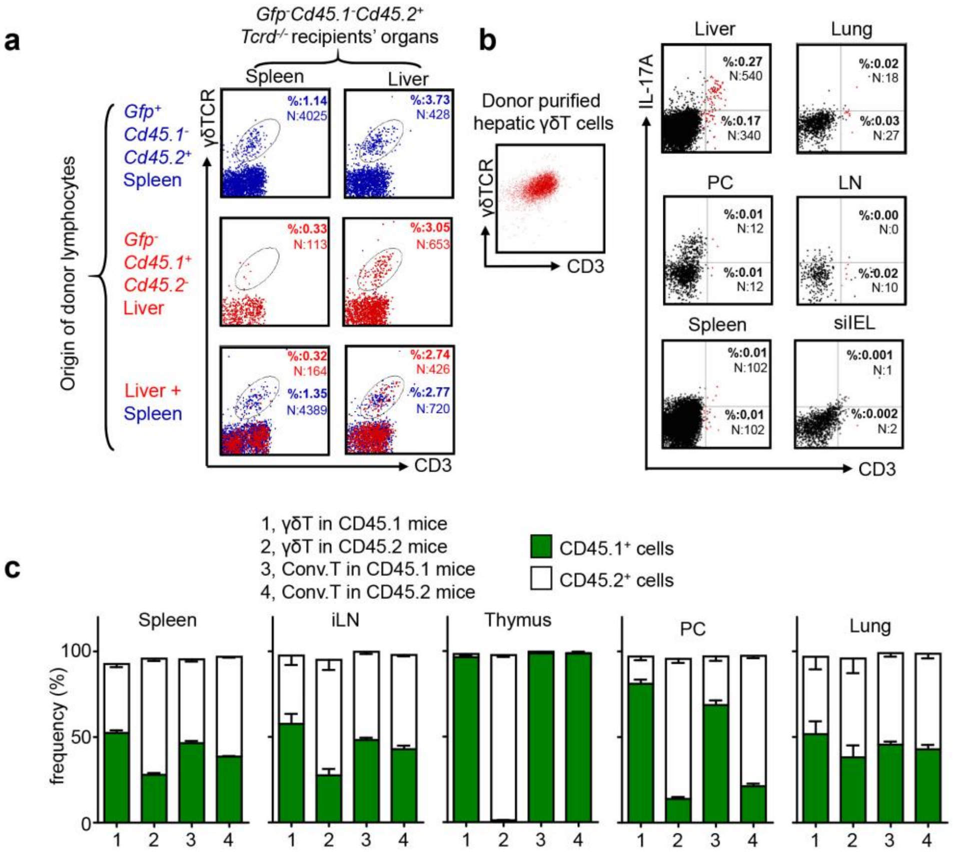

Supplementary Figure 1: Traffic and residency of γδT cells, related to Figure 1. (**a**) *Gfp*$^{+}$*Cd45.1*$^{-}$*Cd45.2*$^{+}$ splenic and *Gfp*$^{-}$*Cd45.1*$^{+}$*Cd45.2*$^{-}$ hepatic lymphocytes (3×10^{6}) were intravenously (*i.v.*) transferred into *Gfp*$^{-}$*Cd45.1*$^{-}$*Cd45.2*$^{+}$ *Tcrd*$^{-/-}$ recipient mice either separately or together. The number (N) and frequency (%) of γδT cells among all the lymphocytes from the same donor (blue for *Gfp*$^{+}$*Cd45.1*$^{-}$*Cd45.2*$^{+}$ splenic cells, red for *Gfp*$^{-}$*Cd45.1*$^{+}$*Cd45.2*$^{-}$ hepatic cells) were evaluated by FACS 24 hours later. (**b**) MACS-purified hepatic γδT cells (3×10^{5}) were *i.v.* transferred into *Rag1*$^{-/-}$ mice, and the γδT-17 cell ($CD3^{+}$γδTCR^{+}IL-$17A^{+}$) numbers (N) and frequency (%) within total lymphocytes ($CD45^{+}$) in the recipient organs were detected by FACS 24 hours later. (**c**) The host origin ($CD45.1^{+}$ or $CD45.2^{+}$) of γδT ($CD3^{+}$TCRγδ^{+}) and conventional T ($CD3^{+}$TCRγδ^{-}NK1.1^{-}) cells was identified by FACS analysis in each mouse of the CD45.1/CD45.2 parabiotic B6 mouse pairs at 14 days post-surgery (n=4 pairs). The data are representative of 3 independent experiments. Either a representative plot or the mean ± SEM is shown (**P* < 0.05; ***P* < 0.01, ****P* < 0.001).

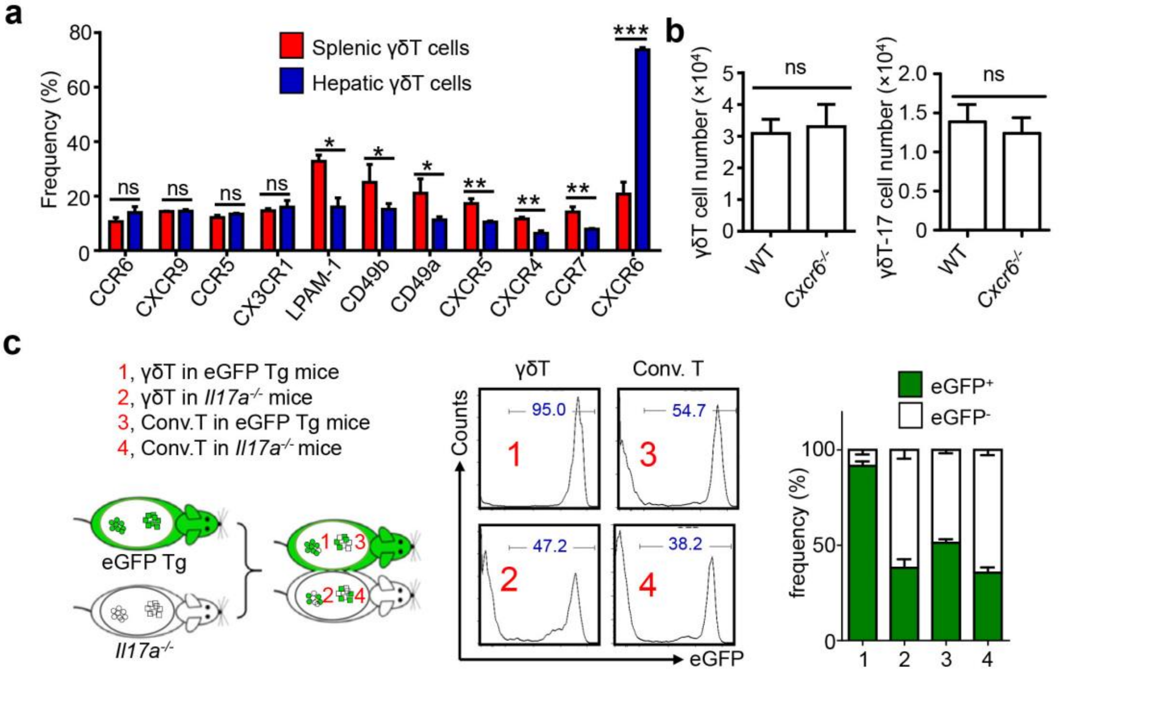

Supplementary Figure 2: Chemokine receptor profile of hepatic γδT cells, related to Figure 1. (**a**) FACS analysis of homing-associated molecules expressed on γδT cells from the liver and spleen. CXCR6 was indicated by GFP expression using CXCR6$^{gfp/+}$ mice (n=3/group). (**b**) The hepatic γδT cell number and γδT-17 cell number in WT mice and *Cxcr6*$^{-/-}$ mice were detected by FACS (n=5/group). (**c**) The host origin (eGFP^{+} or eGFP^{-}) of hepatic γδT (CD3^{+}TCRγδ^{+}) and conventional T (CD3^{+}TCRγδ$^{–}$NK1.1$^{–}$) cells was identified by FACS analysis in each mouse of the eGFP Tg/*Il17a*$^{-/-}$ parabiotic B6 mouse pairs at 14 days post-surgery (n=4 pairs). The data are representative of 3 independent experiments. Either a representative plot or the mean ± SEM is shown (*P < 0.05; **P < 0.01, ***P < 0.001).

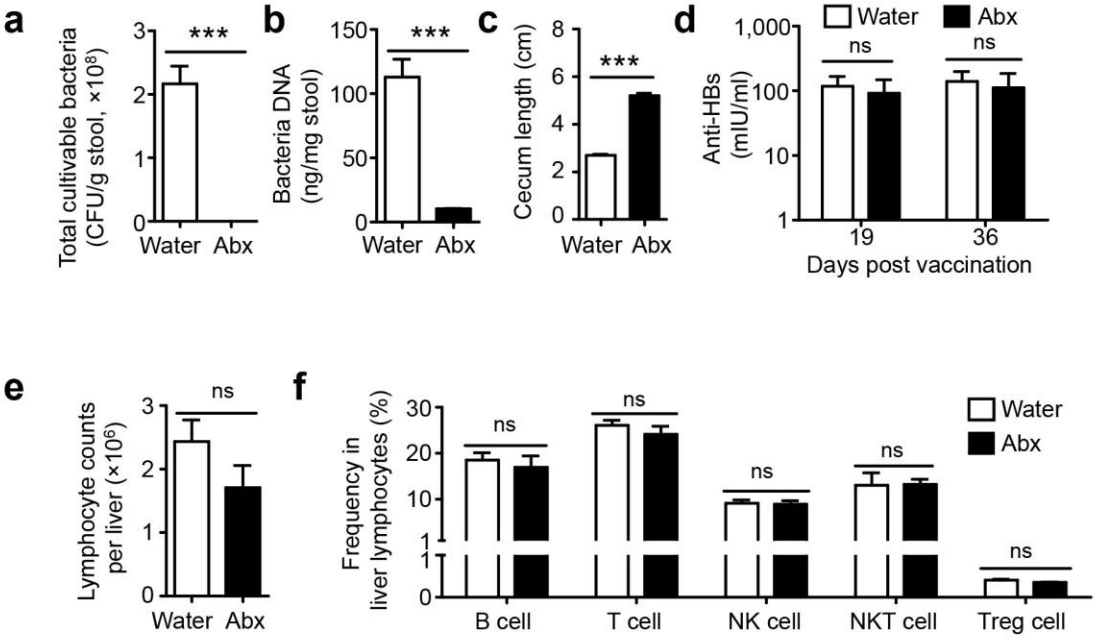

Supplementary Figure 3. Mouse model of antibiotic-mediated commensal clearance, related to Figure 2. Five-week-old B6 mice were fed water alone (Water) or containing antibiotics (Abx) for 4 weeks. (**a**) Total cultivable bacteria in fresh stool were planted and counted on blood agar plates. (**b**) Global stool bacterial DNA was extracted and calculated. (**c**) The cecum length was evaluated. (**d**) Mice were immunized with 50 μg of the HBsAg vaccine twice at 2-week intervals, and serum anti-HBs levels were detected by radioimmunoassay (RIA) at 19 and 36 days post-vaccination. (e) The total number and (**f**) composition of hepatic lymphocytes were evaluated by FACS (B, $CD3^{-}CD19^{+}$; T, $CD3^{+}NK1.1^{-}TCR\gamma\delta^{-}$; NK, $CD3^{-}NK1.1^{+}$; NKT, $CD3^{+}NK1.1^{+}TCR\gamma\delta^{-}$; Treg, $CD3^{+}CD4^{+}Foxp3^{+}$). The data are representative of 3 independent experiments and presented as the mean ± SEM (n = 5/group) (***P* < 0.01, ****P* < 0.001).

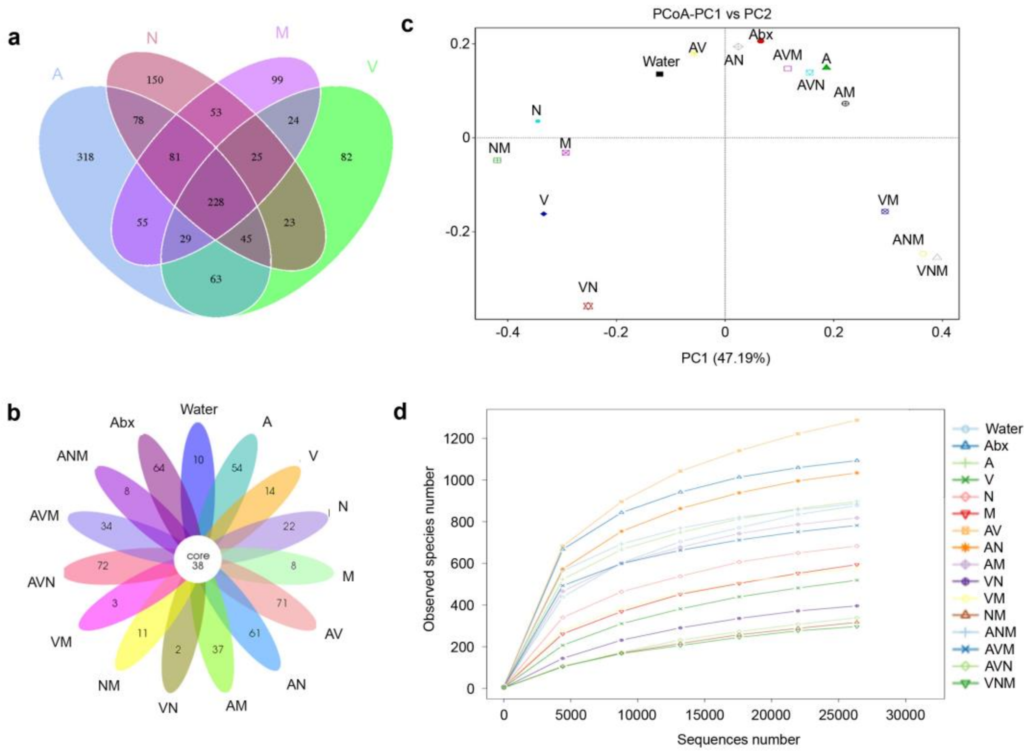

Supplementary Figure 4. Commensal characteristics of mice treated with different antibiotics, related to Figure 3. Mice were treated as described in Figure 3. Genomic bacterial DNA was isolated from fecal samples, and the V5-V6 hypervariable region of 16S rDNA was amplified and sequenced. (**a, b**) Venn diagram (**a**) or Petal diagram (**b**) showing the overlap and specificity of bacterial species in different antibiotic-treated mice. The number of OTU reads is shown. (**c**) Discrimination of mouse groups based on microbial composition. Scatter plot scores of the principal coordinate analysis (PCoA) of microbial profiles are shown. (**d**) The rarefaction curve of the OTUs in each group.

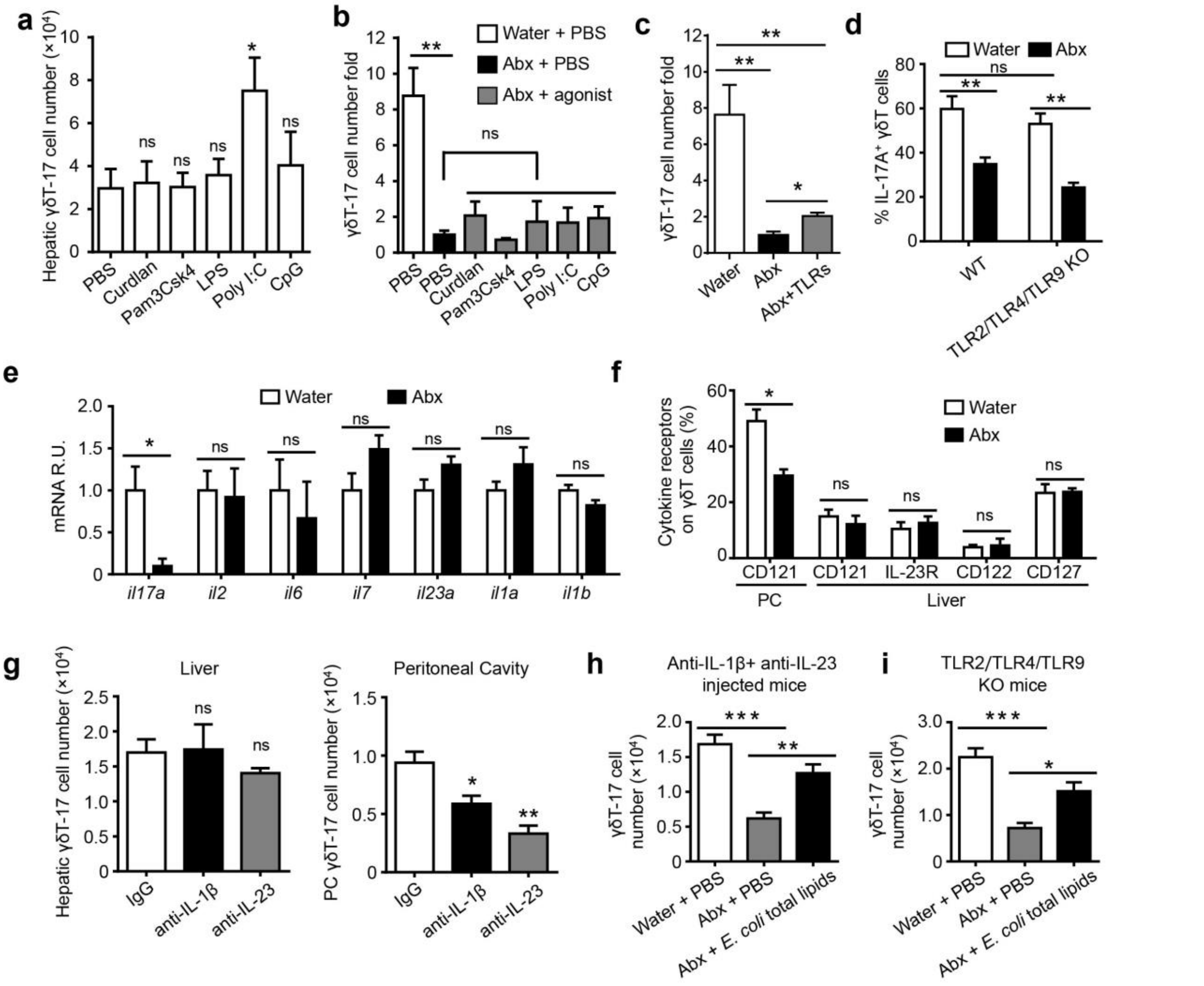

Supplementary Figure 5. The reduction of hepatic γδT-17 cells in Abx-treated mice is independent of PAMPs and cytokine signals, related to Figure 5. Five-week-old B6 mice were fed water alone (Water) or containing antibiotics (Abx) for 4 weeks. (**a, b**) Water-fed mice (**a**) and Abx-treated mice (**b**) were *i.p.* injected with 50 µg curdlan, 50 µg Pam3csk4, 100 µg LPS, 100 µg poly(I:C) or 50 µg CpG. Hepatic γδT-17 cells were evaluated by FACS one day later, and the cell numbers (**a**) and fold increase of the cell number compared with Abx-treated mice receiving PBS (**b**) are shown (n=4/group). (**c**) Abx-treated mice were *i.v.* injected with 50 µg Pam3csk4, 10 µg LPS and 50 µg CpG 3 times on days 0, 3 and 6. Hepatic γδT-17 cells were evaluated by FACS on day 8, and the cell number fold change compared with Abx-treated mice receiving PBS is shown (n=5, 4, 5). (**d**) Co-housed WT mice and TLR2/TLR4/TLR9 triple-knockout mice were treated with antibiotics for 4 weeks, and the frequency of hepatic γδT-17 cells was evaluated by FACS (n=5/group). (**e**) *Il17a*, *Il2*, *Il6*, *Il7*, *Il23*, *Il1a* and *Il1b* mRNA expression in the livers of Water- and Abx-treated mice were evaluated by RT-PCR (n=5, 4.). (**f**) IL-23R, CD122, CD127 and CD121 expression levels on liver and PC γδT cells from WT and Abx-treated mice were evaluated by FACS (n=5,4.). (**g**) B6 mice were *i.v.* injected with 50 µg control IgG, anti–IL-1β or anti–IL-23 antibody twice at 3-day intervals, and hepatic and PC γδT-17 cells were analyzed by FACS 3 days after the last injection (n=4,5,4.). (**h, i**) Antibody-injected mice (50 µg anti–IL-1β and 50 µg anti–IL-23, once per week) (**h**) and TLR2/TLR4/TLR9 triple-knockout mice (**i**) were treated with antibiotics or untreated, and Abx-treated mice were then i.p. injected with 50 µg total *E. coli* polar lipid extract 6 times at 2-day intervals. The hepatic γδT-17 cell numbers were evaluated by FACS (n=4/group). The data are representative of 3 independent experiments and are presented as the mean ± SEM (*P < 0.05; **P < 0.01).

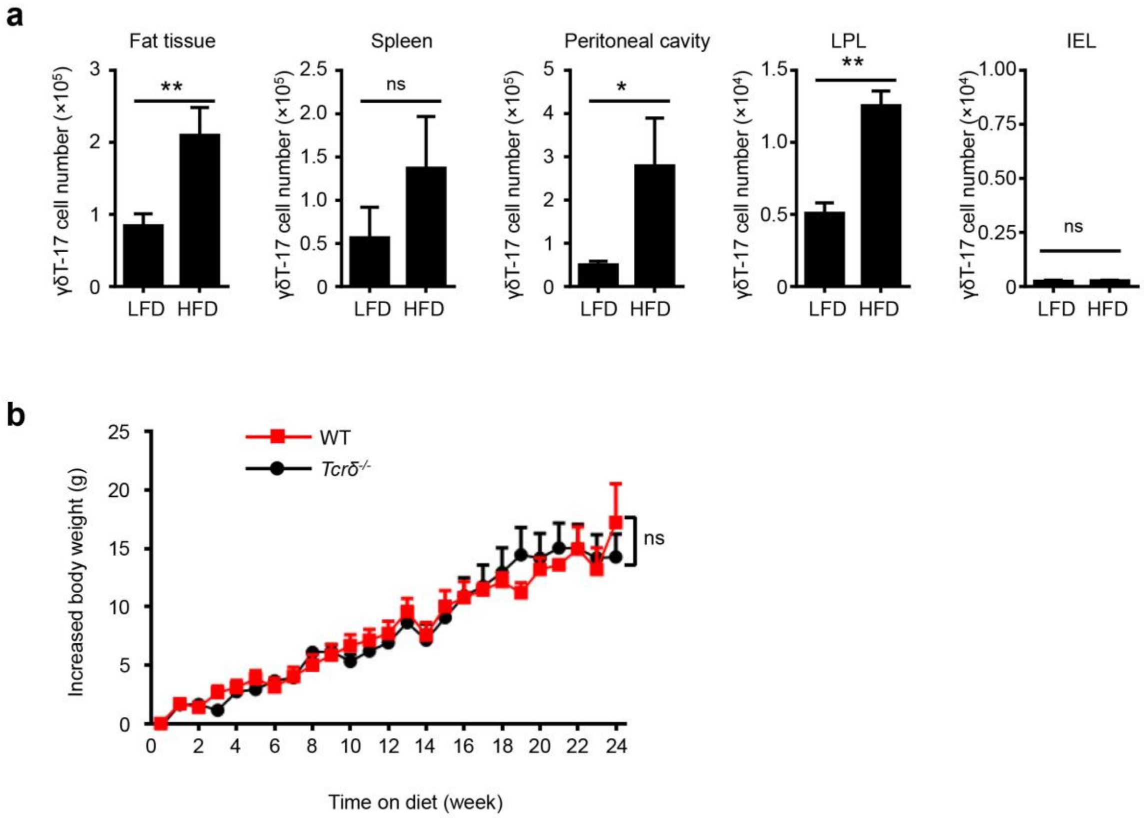

Supplementary Figure 6. γδT-17 cell accumulation and body weight increase of HFD mice, related to Figure 7. WT and *Tcrδ*$^{-/-}$ mice were placed on an HFD for 24 weeks. (**a**) The γδT-17 cell numbers in the fat tissue, spleens, peritoneal cavities, lamina propria lymphocytes (LPLs) and intraepithelial lymphocytes (IELs) were detected by FACS (n=4, 5). (**b**) Increased body weight of HFD-fed WT mice and *Tcrδ*$^{-/-}$ mice (n=5/group). The data are representative of 3 independent experiments and shown by the mean ± SEM (*p < 0.05; **p < 0.01).

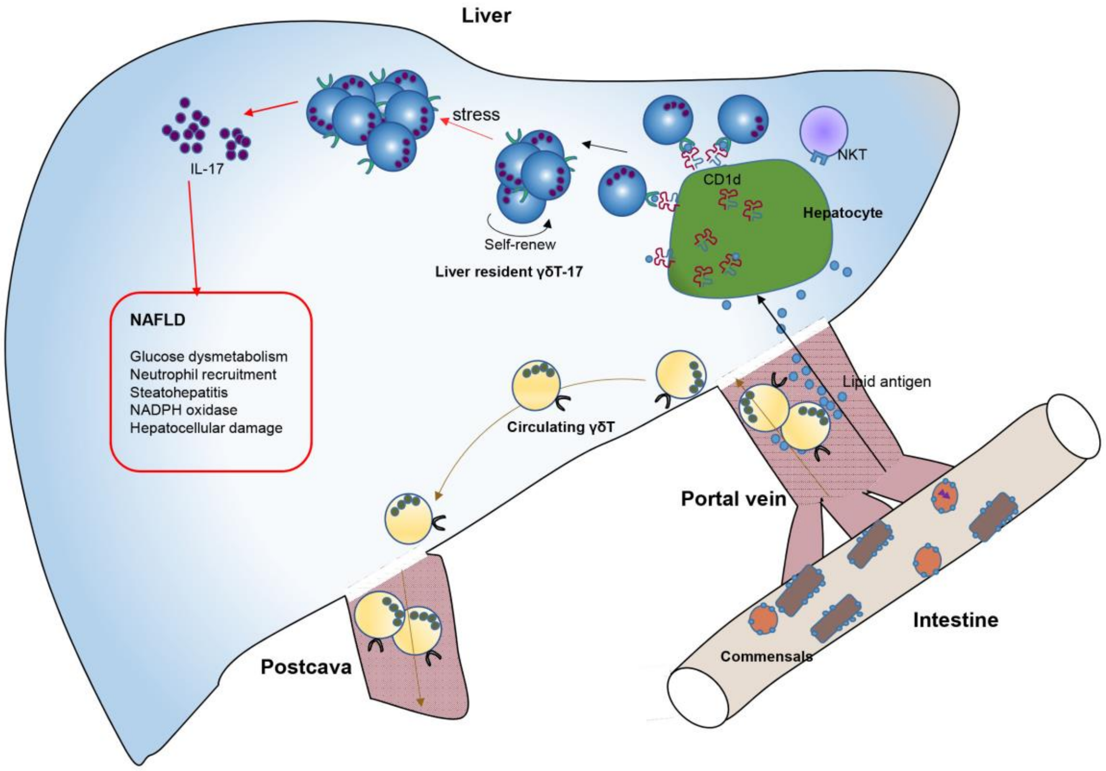

Supplementary Figure 7. Schematic diagram of microbiota-promoted liver-resident γδT-17 cells. Microbiota/*E. coli* lipid antigens reach the liver through the portal vein. Then, they will be presented by CD1d expressed on hepatocytes to γδTCR, which induces the activation, anti-apoptosis and proliferation of hepatic γδT cells. These self-renewing hepatic γδT cells are resident in the liver and cannot be replaced by circulating γδT cells. The liver-resident γδT cells predominantly express IL-17A. More importantly, under stress conditions, liver-resident γδT-17 cells expend and produce high levels of IL-17A, which then accelerate NAFLD. If the microbiota are depleted, liver-resident γδT-17 cells sharply reduce and skew the outcome of this pathological process. Thus, liver-resident γδT-17 cells link the microbiota to shape the liver immune response.

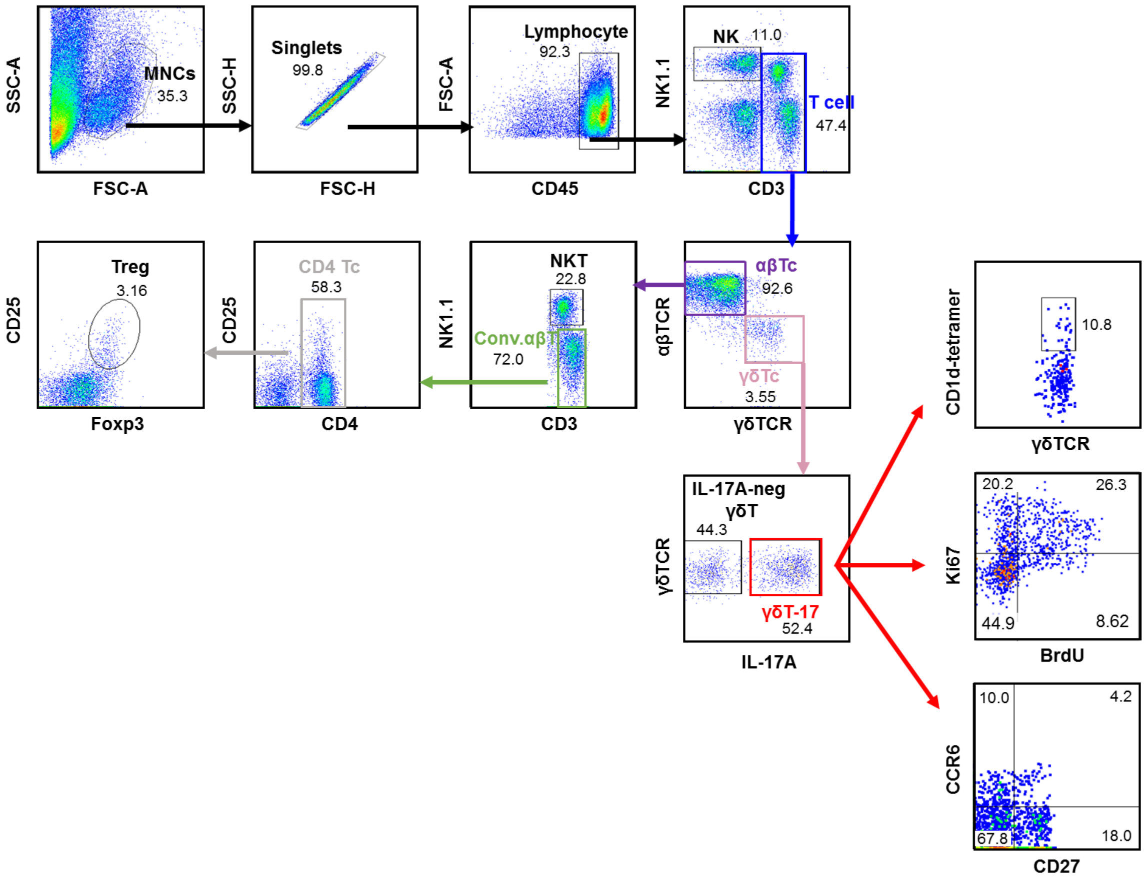

Supplementary Figure 8. Flow cytometry gating strategy, related to Methods. MNCs, mononuclear cell; NK, natural killer cell; αβTc, αβT cell; γδTc, γδT cell; NKT, natural killer T cell; Conv. αβTc, conventional αβT cell; CD4 Tc, CD4 T cell; Treg, regulatory T cell; γδT-17, IL-17 secreted γδT cells.

Supplementary Table 1. Anti-mouse monoclonal antibodies for flow cytometry and *in vivo* neutralization.

**Fluorescence**	**Antigen**	**Clone**	**Company**	**Catalog number**	**Isotype control**	**Dilution**
FITC	CD19	1D3	BD	557398	Rat IgG2a, κ	1:200
FITC	CD25	7D4	BD	553072	Rat IgM, κ	1:200
FITC	CD27	LG.7F9	eBioscience	11-0271	ArH IgG	1:200
FITC	CD62L	MEL-14	BD	553150	Rat IgG2a, κ	1:200
FITC	TCRγδ	GL3	eBioscience	11-5711	ArH IgG	1:200
FITC	CD122	TM-β1	BD	553361	Rat IgG2b, κ	1:100
FITC	CD8α	53-6.7	BD	553031	Rat IgG2a, κ	1:200
FITC	CXCR4	2B11	BD	551967	Rat IgG2b, κ	1:200
FITC	CD49b	DX5	BD	553857	Rat IgM, κ	1:200
PE	Biotin	ID4-C5	BioLegend	409003	Ms IgG2a, κ	1:200
PE	CD4	RM4-5	BD	553049	Rat IgG2a, κ	1:100
PE	CD45.1	A20	BD	553776	Ms IgG2a, κ	1:200
PE	IL-17	TC11-18H10	BD	559502	Rat IgG1, κ	1:200
PE	TCRγδ	GL3	BD	553178	ArH IgG2, κ	1:200
PE	CD121	35F5	BD	557489	Rat IgG1, κ	1:200
PE	CD127	SB/199	BioLegend	121111	Rat IgG2b, κ	1:200
PE	CCR6	29-2L17	BioLegend	129804	ArH IgG	1:100
PE	TCRVγ2	UC3-10A6	BioLegend	137706	ArH IgG	1:200
PE	CCR5	HM-CCR5	BioLegend	107005	ArH IgG	1:200
PE	CCR9	9B1	BioLegend	129705	Rat IgG2a, κ	1:200
PE	CX3CR1	SA011F11	BioLegend	149005	Ms IgG2a, κ	1:200
PE	CD49a	Ha31/8	BD	562115	ArH IgG2,λ1	1:200
PE	LPAM-1	DATK32	eBioscience	12-5887	Rat IgG2a, κ	1:200
PerCp-CY5.5	Foxp3	FJK-16s	eBioscience	45-5773	Rat IgG2a, κ	1:200
PerCp-CY5.5	CD3e	145-2C11	BD	551163	ArH IgG1, κ	1:200
PerCp-CY5.5	IL-17a	TC11-18H10.1	BioLegend	506920	Rat IgG1, κ	1:200
PerCp-CY5.5	CD127	A7R34	BioLegend	135021	Rat IgG2a, κ	1:200
PerCp-CY5.5	CD24	M1/69	BioLegend	101823	Rat IgG2b, κ	1:200
PerCp-CY5.5	CD45.2	104	BD	552950	Ms IgG2a, κ	1:200
PerCp-CY5.5	CXCR5	L138D7	BioLegend	145508	Rat IgG2b, κ	1:200
PerCp-CY5.5	CCR7	4B12	eBioscience	45-1971	Rat IgG2a, κ	1:200
PE-CY7	NK1.1	PK136	BD	552878	Ms IgG2a, κ	1:200
PE-CY7	CD45.1	A20	BD	560578	Ms IgG2a, κ	1:200
PE-CY7	IFN-γ	XMG1.2	BD	557649	Rat IgG1, κ	1:400
APC	Gr-1	RB6-8C5	BD	553129	Rat IgG2b, κ	1:200
APC	TCRVγ1.1	2.11	BioLegend	141108	ArH IgG	1:200
APC	TCRγδ	eBioGL3	eBioscience	17-5711	ArH IgG	1:200
Alexa 647	IL-17A	TC11-18H10	BD	560184	Rat IgG1, κ	1:200
Alexa 647	Ki67	SolA15	eBioscience	51-5698	Rat IgG2a	1:400
APC-CY7	CD3e	145-2C11	BD	557596	AH IgG1, κ	1:200
APC-CY7	CD4	GK1.5	BD	552051	Rat IgG2b, κ	1:200

APC-CY7	CD11b	M1/70	BD	557657	Rat IgG2b, κ	1:200
Functional	IL-1β	B122	BioLegend	503504	ArH IgG	NA
Functional	IL-23(P19)	MMp 19B2	BioLegend	513806	Ms IgG2b, κ	NA
Functional	ArH IgG	HTK888	BioLegend	400916	ArH IgG	NA

Supplementary Table 2. Primers for RT-PCR.

**Gene**	**Forward (5′-3′)**	**Reverse (5′-3′)**
il17a	GGCTGACCCCTAAGAAACC	CTGAAAATCAATAGCACGAAC
il2	AACCTGAAACTCCCCAGGAT	TCATCGAATTGGCACTCAAA
il6	AACGATGATGCACTTGCAGA	GGAAATTGGGGTAGGAAGGA
il7	AGAGTGTACTGATGATCAGC	GCAGTTCACCAGTGTTTGTG
il23a	CCAGCGGGACATATGAATCT	AGGCTCCCCTTTGAAGATGT
il1a	GCAACGGGAAGATTCTGAAG	TGACAAACTTCTGCCTGACG
il1b	GACCTTCCAGGATGAGGACA	AGGCCACAGGTATTTTGTCG
actin	TGACGTTGACATCCGTAAAGACC	CTCAGGAGGAGCAATGATCTTGA
p67phox	*CTATCAGCTGGTTCCCACGA*	*GCAGTGGCCTACTTCCAGAG*
p47phox	*ATGACCTCAATGGCTTCACC*	*CTATCTGGAGCCCCTTGACA*
p22phox	*CCTGCSGCGATAGAGTAGGC*	*TCATGGGGCAGATCGAGT*
Nox2	*CGGTGTGCAGTGCTATCATC*	*GCTCTCCTTTCTCAGGGGTT*
